# Agriculture without paraquat is feasible without loss of productivity—lessons learned from phasing out a highly hazardous herbicide

**DOI:** 10.1007/s11356-022-24951-0

**Published:** 2023-01-09

**Authors:** Alexander M. Stuart, Charles N. Merfield, Finbarr G. Horgan, Sheila Willis, Meriel A. Watts, Fernando Ramírez-Muñoz, Jorge Sánchez U, Leah Utyasheva, Michael Eddleston, Mark L. Davis, Lars Neumeister, Manoé R. Sanou, Stephanie Williamson

**Affiliations:** 1 Pesticide Action Network UK, Brighthelm Centre, Brighton, UK; 2 Merfield Agronomy Ltd, Lincoln, New Zealand; 3 Centre for Pesticide Suicide Prevention, University of Edinburgh, Edinburgh, UK; 4 Facultat de Ciencias Agrarias Y Forestales, Escuela de Agronomía, Universidad Católica del Maule, Casilla 7-D, 3349001 Curico, Chile; 5 EcoLaVerna Integral Restoration Ecology, Bridestown, Kildinan, T56 P 499 Cork Ireland; 6 PAN Asia Pacific, Simpang Ampat, Penang, Malaysia; 7 Central American Institute for Studies On Toxic Substances (IRET), Universidad Nacional, Heredia, Costa Rica; 8 Nicoverde S.A, Alajuela, Costa Rica; 9 Backnang, Germany; 10 Department of Plant Protection and Packaging, Ministry of Agriculture, Ouagadougou, Burkina Faso

**Keywords:** Agricultural policy, Agroecology, Highly hazardous pesticides, Integrated weed management, Pesticide poisoning, Pesticide regulation, Sustainable crop production

## Abstract

A small proportion of the thousands of pesticides on the market today are associated with a disproportionately high incidence of severe acute pesticide poisoning and suicide. Paraquat stands out as one of the most lethal pesticides in common use, frequently involved in fatal incidents due to suicides or accidental exposure. Even though paraquat has been banned in over 67 countries, it is still widely used in many others, particularly in Asia and Latin America. Based on a literature review and consultations, this paper identifies options for replacing paraquat and distils practical lessons from numerous successes around the world. Our aim is to support regulators, policymakers, agronomists and the supply chain sector with practical information related to phasing out paraquat*.* Production data consistently failed to show any negative effects of banning paraquat on agricultural productivity. A wide range of alternative approaches to weed management and crop defoliation are available, many of which do not rely on herbicides. Over 1.25 million farmers in low- and middle-income countries (LMICs) successfully produce a range of crops for private voluntary standards (PVS) in food and fiber supply chains which prohibit paraquat use. We conclude from the findings of this study that eliminating paraquat will save lives without reducing agricultural productivity. Less hazardous and more sustainable alternatives exist. To enhance successful adoption and uptake of these methods on a wide scale, farmers require training and support within an enabling policy environment.

**Supplementary Information:**

The online version contains supplementary material available at 10.1007/s11356-022-24951-0.

## Introduction

Paraquat (1,1′-dimethyl-4,4′-bipyridylium) is a contact herbicide first developed for commercial purposes in the 1950s (Brian et al. 1958). It is commonly used in many countries due to its low-cost and broad-spectrum efficacy (Neumeister and Isenring 2011). However, increasing evidence demonstrates a high acute risk of paraquat to human health due to its high toxicity. For example, it is one of the most frequently used pesticides in suicides (Chang et al. 2022), a consequence of its availability in many rural areas and high case fatality (Kim and Kim 2019). The lethal dose for adults by ingestion is only 35 mg/kg b.w., which is less than a mouthful of a 20% solution, and the case fatality following intentional ingestion is as high as 80% due to the lack of an effective treatment (Flechel et al. 2018; Proudfoot et al. 1979; Raghu et al. 2013).

Suicide is one of the leading causes of human mortality worldwide, with more people dying from suicide than HIV, malaria, war, or homicide (WHO 2021a). It is particularly problematic in low- and middle-income countries (LMICs), and it is the fourth leading cause of death among 15–29 year olds. Pesticide self-poisoning currently accounts for 15–20% of all suicides, with an estimated 110,000–168,000 pesticide suicides each year (Mew et al. 2017). Since the Green Revolution started in the 1950s, an estimated 14 million people have died from pesticide self-poisoning (Karunarathne et al. 2020). In 2017, pesticide poisoning was the leading method for suicide in China (Page et al. 2017) and in 2010, S. Korea recorded 3206 suicides following ingestion by paraquat, before it was banned there (Kim and Kim 2019). Paraquat is also involved in fatal and non-fatal unintentional poisonings, including in countries with high use of protective equipment and mitigation measures. For example, the U.S. Environmental Protection Agency (EPA) revealed that out of 27 paraquat fatality reports during 2014, eight were due to the accidental ingestion of paraquat (US EPA 2021a). In Malaysia, paraquat was responsible for 45% of 2,226 reported pesticide poisoning cases from 1997 to 2009 due to accidental ingestion and occupational exposure as well as from self-harm and suicides (CRC 2021).

Cases of intentional and unintentional pesticide poisoning are severely under-reported (Kamaruzaman et al. 2020; Litchfield 2005). A systematic review by Boedeker et al. (2020) estimated that approximately 385 million cases of unintentional, acute pesticide poisoning occur annually worldwide, a far higher number than previous estimates. Acute health effects from paraquat include eye injury, nosebleeds, skin irritations, and burns. Sub-lethal doses or absorption via dermal exposure can cause severe damage to the lungs and kidneys (Isenring 2017, Neumeister and Isenring 2011). Paraquat is also implicated in chronic health effects: for example, a recent meta-analysis confirmed an association between paraquat use and Parkinson’s disease (Tangamornsuksan et al. 2019).

Paraquat also poses a risk to the environment (Cousin et al. 2013; Rosic et al. 2020; Sartori et al. 2018). It is moderately to highly toxic to mammals, birds, and aquatic invertebrates. According to the US EPA (2019a), it is “very persistent in soil/sediment and accumulates in the environment in an adsorbed state.” Upon exposure to soil moisture or water, paraquat dichloride loses the negatively charged chloride ions and rapidly and almost completely adsorbs to soil particles and/or sediment. Because of this rapid adsorption, soil microorganisms degrade less than 1% of paraquat (Roberts et al. 2002), making it extremely persistent, with a half-life (DT50) in field conditions of at least seven years (US EPA 2019b). In US EPA laboratory fate studies, DT90 values (i.e., 90% degradation) were never reached. Although desorption from soil particles is not expected and was not detected in laboratory studies, questions remain about the potential for toxic exposures through desorption in the digestive tracts of sediment ingesting epibenthic and infaunal detritivores (US EPA 2019a). Additionally, a trial using vineyard soils in Spain found that while 70–90% of paraquat was adsorbed to soils, 11% was desorbed again (Pateiro-Moure et al. 2009). Furthermore, paraquat may be vertically transported through the soil profile to water bodies via eroded soil particles, with paraquat residues previously detected in ground and surface waters in Thailand (Amondham et al. 2006; Vinten et al. 1983). Thus, paraquat use can lead to long-term contamination of both soils and aquatic environments (Huang et al. 2019; Pateiro-Moure et al. 2009).

**Fig. 1:**
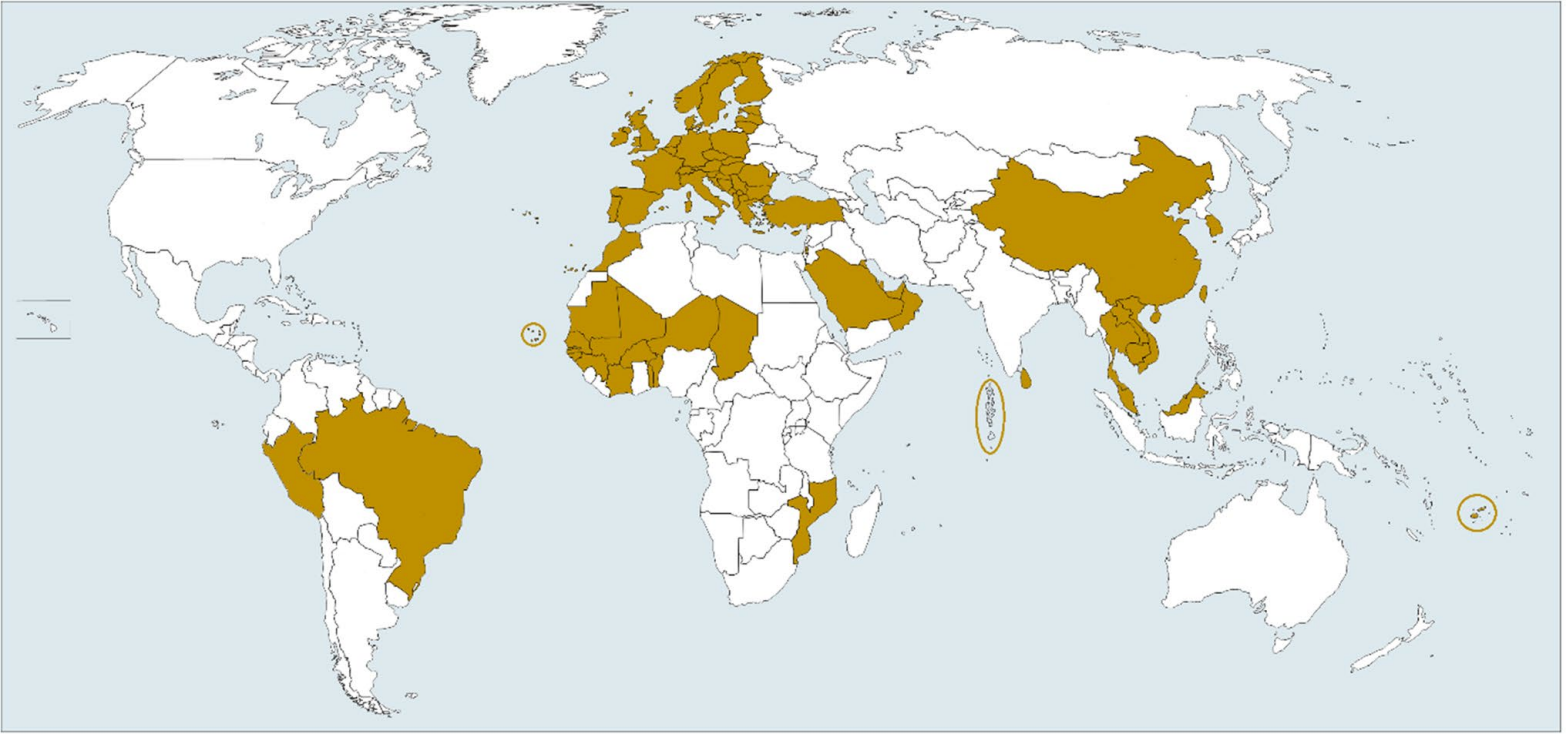
Nations (shaded in brown) that currently ban or severely restrict use of paraquat. The full list of the countries and the years in which paraquat bans were implemented (along with known phase out periods) are provided in Supplementary Table S1

In response to known harms, particularly to human health, over 67 countries have banned the use of paraquat (see Fig. 1; Supplementary Table S1), and many private voluntary standards (PVS, also known as voluntary sustainability standards) in certified food and fiber supply chains and retailer companies have included paraquat in their prohibited chemical lists. In 2011, the Rotterdam Convention Chemicals Review Committee (CRC) recommended herbicide formulations containing > 200 g/L paraquat for listing in Annex III of the Convention, meaning that it was agreed that paraquat met the Annex III criteria for chemicals that have been banned or severely restricted for health or environmental reasons by two or more parties (CRC 2011). This expert review included evidence of occupational exposure incidents of paraquat poisoning in Burkina Faso and many other countries. However, a small minority of countries (i.e., India, Argentina, Paraguay, Guatemala, Iran, and Indonesia in 2022) have opposed the listing of paraquat in Annex III (IISD 2022). By doing so, they prevented importing countries around the world from using the Prior Informed Consent Procedure for paraquat, which is designed to help them to better control imports of this hazardous chemical.

In some countries, paraquat use has been restricted but not banned. For example, the U.S. EPA recently implemented regulations that included the prohibition of backpack sprayers and restricting use to certified pesticide applicators only (US EPA 2021b). However, the efficacy of such restrictions is questionable and would be especially difficult to implement in many LMICs due to factors such as limited resources for training and enforcement, current dependence on backpack sprayers for application, improper labeling, low-level literacy, and protective clothing that is scarce, too expensive, and/or inappropriate for hot climatic conditions (Zaller 2020).

As there is no antidote for paraquat poisoning, it is recommended that the main focus should be on preventive measures, in particular, elimination—the most effective and highest level of the industrial Hierarchy of Control (Knipe et al. 2017; Raghu et al. 2013; WHO 2021b, a). There is clear evidence that suicide numbers decrease following bans. For example, following paraquat bans, pesticide-related suicides in South Korea, Sri Lanka, and Taiwan fell by 46, 50, and 37%, respectively, and overall suicides in S. Korea and Sri Lanka fell by 17 and 21%, respectively (Chang et al. 2022; Kim and Kim 2019; Knipe et al. 2017). In Malaysia, the number of emergency medical calls relating to paraquat exposure more than doubled in 2007 compared to 2006, when the paraquat ban was lifted following a two-year ban, and it rose by 5.5 times from 2006 to 2015 (Leong et al. 2018; Sazaroni et al. 2012). Paraquat was subsequently banned again in 2020. The direct relation between banning of highly hazardous pesticides (HHP) such as paraquat and consequent reductions in suicides is because decisions to self-harm generally occur impulsively (often occurring after less than 30 min of thought), and survival provides individuals in distress the time for acute crises to pass and for them to receive support (WHO 2021b).

Based on a literature review and consultation process, this paper identifies alternative approaches to replace paraquat and to distil practical and policy lessons from the numerous successes in phasing out paraquat worldwide. This will provide regulators, policymakers, agronomists, and the supply chain sector with information to support phasing out paraquat. Specifically, the paper covers the following topics: the uses of paraquat; the effects of national paraquat bans on agricultural production; alternative weed management approaches to replace paraquat; case studies of successful alternative approaches; and key lessons that emerged from a consultation of pesticide regulators, PVS organisations, and UK retailers.

## Paraquat—why is it used?

Paraquat (1,1′-dimethyl-4,4′-bipyridylium) belongs to the small group of bipyridylium herbicides that are quaternary ammonium salts known as “quats,” which also include the herbicide diquat (1,1′-ethylene-2,2′-bipyridylium). The term “paraquat” has been applied to three technical products: paraquat, paraquat dichloride (1,1′-dimethyl-4,4′-bipyridylium dichloride), and paraquat dimethylsulfate (1,1′-dimethyl-4,4′-bipyridylium dimethylsulfate) (WHO, 1984). The term “paraquat” is generally used to cover all three of these, but most paraquat formulations contain paraquat dichloride.

Paraquat is a broad spectrum, non-systemic, non-selective herbicide which kills all green plant material it is applied to. It is described as a “burn-down” herbicide. Plant tissue is destroyed via the disruption of photosynthesis and rupturing of cell membranes that allows water to escape, causing the foliage to desiccate (Watts 2011). However, it does not kill plants that can regenerate from protected meristems, such as underground crowns or rhizomes, and it does not penetrate the bark of trees. Due to its burn down action, paraquat has been used to clear land of weeds before planting, including in no-till systems, and to remove weeds between crops after emergence, especially for fruit and plantation crops (e.g., banana, coffee, oil palm, and tea) and field crops, such as maize (see Table 1). Paraquat is also used for weed control in non-agricultural areas such as roadsides, airports, around commercial buildings and homes, drains, irrigation ditches, and waterways (Watts 2011, Wesseling et al. 2001).

**Table 1:** A summary of the most common uses for paraquat and the main agricultural crops it is used for (sources: Bellaloui et al. (2020), Griffin et al. (2010), Neumeister and Isenring (2011), UNEP (2012), Wesseling et al. (2001), Zaller (2020))

Common uses	Reasons	Main agricultural crops
Pre-planting weed control	Low cost; labor-saving vs. manual only; glyphosate weed resistance	Maize; soybean; rice
Post-emergent weed control	Low cost; labor-saving vs. manual only	Banana; coffee; oil palm; orchard crops; tea
Pre-harvest defoliant	Improve harvest efficiency; reduce crop damage during harvest process	Cotton; potato
Pre-harvest desiccant	Improve harvest efficiency; shorten harvest to replanting interval	Soybean
Post-harvest desiccant	To remove breeding habitat for stable fly; shorten harvest to planting interval	Pineapple; soybean

In addition to weed management, paraquat is widely used as a desiccant to remove crop foliage before harvest (Griffin et al. 2010). It is applied directly to the crop prior to harvest to either accelerate seed maturity and crop uniformity (e.g., soybean), as a “harvest-aid” to facilitate mechanical harvesting by removing green foliage (e.g., cotton and potato), or to increase sucrose concentration in sugarcane (Zaller 2020). However, pre-harvest use of paraquat for desiccation is particularly controversial because it increases the risk of residues in food products, despite mandatory intervals between spraying and harvesting if these are implemented (Beckie et al. 2020). If paraquat is applied before physical maturity, it can lead to reductions in yield and seed nutritional quality (Bellaloui et al. 2020). In intensified cropping systems, paraquat is used as a desiccant to remove crop residues after the crop is harvested, which can reduce the interval between harvest and planting of the next crop. It is also used to destroy pineapple foliage which is a breeding habitat for stable fly, *Stomoxys calcitrans*, an important pest of livestock in Costa Rica (UNEP 2012).

Recently, paraquat has also been employed to combat weeds resistant to glyphosate, especially in glyphosate-tolerant crops where multiple applications of glyphosate per cropping season have led to the emergence of glyphosate-resistant weed populations. To manage glyphosate weed resistance, paraquat (or diquat) is applied prior to crop sowing along with glyphosate as part of the double-knock technique (Beckie et al. 2020; Walsh and Kingwell 2021). This has led to an increase in paraquat use, as well as overall herbicide use in glyphosate-tolerant crops in comparison with non-glyphosate-tolerant crops (Neumeister 2016, Perry et al. 2016). However, due to its already common and widespread use, the evolution of weed resistance to paraquat is also a major concern. Paraquat is currently 10th among the top 15 herbicide active ingredients for which weed resistance is documented, with resistance documented in 31 species of weeds (Heap 2022; Supplementary Table S2). In addition, several cases of multiple resistances to both paraquat and glyphosate have emerged (Brunharo and Hanson 2018; Jalaludin et al. 2015; Moretti and Hanson 2017; Moretti et al. 2017; Tehranchian et al. 2018; Yu et al. 2007). The emergence of such multiple-resistant weed populations will severely restrict the possibilities for using paraquat to manage resistance to glyphosate herbicides or vice versa (Dennis et al. 2016; Owen et al. 2014).

## Effects of paraquat bans on agricultural production

Despite awareness of deaths and poisonings associated with paraquat, and bans in over 67 countries, it remains in use in others. A common argument against banning paraquat from use in agriculture is that there will be a negative effect on crop yield, which will in turn reduce farmers’ livelihoods and affect food security. However, an increasing number of studies indicate that paraquat bans do not have negative effects on agricultural productivity.

In a recent study, Sethi et al. (2022) explored potential effects on crop yields in Kerala, India, resulting from a ban of 14 highly hazardous pesticides (HHPs), including paraquat dichloride, in 2011. The study looked at crop production in eight key crops using survey data from the Government of Kerala for 6–7 years on either side of the ban. They found no evidence of effect on agricultural yields in any of the crops studied, and there was no evidence that rainfall may have masked underlying effects of the ban. In addition, a study in South Korea found no evidence that crop yield was affected by the paraquat ban (Kim and Kim 2019). In Taiwan, there also was no obvious change in the yields of four major crops after the paraquat ban (Chang et al. 2022).

To supplement the existing literature investigating the effect of banning paraquat on crop yields, we analyzed crop production data available on FAOSTAT (https://www.fao.org/faostat/). This included aggregated data for all crops reported in FAOSTAT for six CILSS (Permanent Inter-State Committee for Drought Control in the Sahel) member countries in Africa (Fig. 1.), which all banned paraquat in August 2011 (CILSS 2011) and five other countries that also banned paraquat at least six years ago. The selection of the countries was based on the length of time since the paraquat ban and availability of data. A minimum six-year period was chosen to take into account annual fluctuations in yields and time delays for restrictions to enter effect. In each case, we analyzed data from 6 to 8 years either side of the ban. This involved selecting the following filters from the “Crops and Livestock Products” data in FAOSTAT: “countries,” “crops primary,” “production,” “harvested area,” and “years.” Mean annual yield was calculated by dividing the sum of “production” by the sum of “harvested area” for all crops reported per year. We also compiled the FAOSTAT “yield” data for key individual crops in which paraquat was heavily used prior to being banned in the respective country. These are potatoes in UK (https://pusstats.fera.co.uk/) and France (Pouchieu et al. 2018), cotton and maize in Burkino Faso (CRC 2011), and tea in Sri Lanka (Marambe and Herath 2019).

**Fig. 2:**
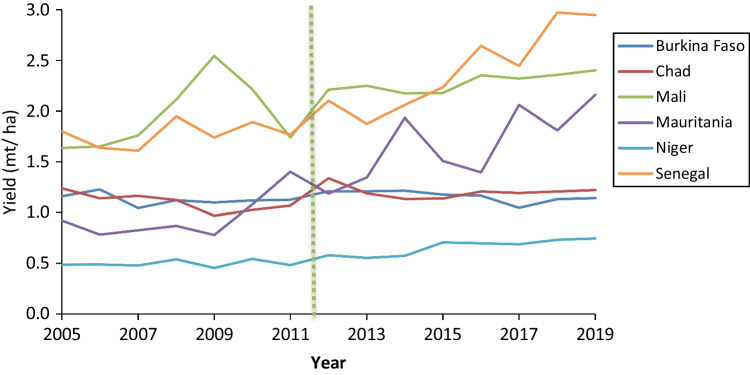
Mean annual yield of all crops reported in FAOSTAT for six West African CILSS countries 6–8 years before and after paraquat was banned in August 2011. Vertical line indicates time of ban (source: FAOSTAT data accessed on 03 February 2022)

**Fig. 3:**
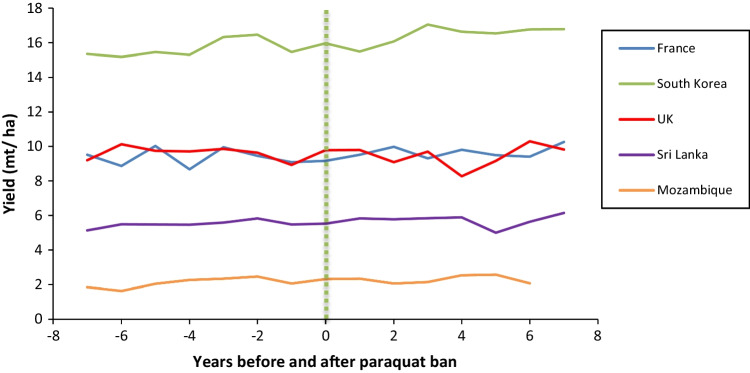
Mean annual yield for all crops reported in FAOSTAT for five countries 6–7 years before and after paraquat was banned. Vertical line indicates year of ban (source: FAOSTAT data accessed on 3.^rd^ February 2022)

**Fig. 4:**
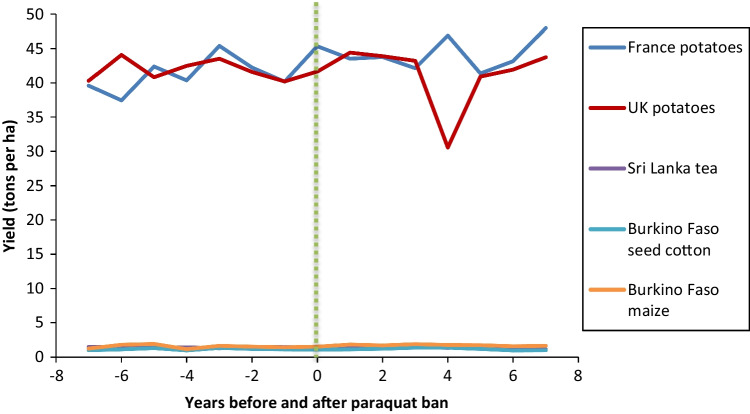
Mean annual yield for previously paraquat dependent crops in five countries 6–7 years before and after paraquat was banned. Vertical line indicates year of ban (source: FAOSTAT data accessed on 03 February 2022) (yields for the lowest yielding country x crop combinations are provided at higher resolution in Supplementary Fig. S1)

In 9 out of the 11 countries studied, there was no observable decline in aggregated crop yields (Figs. 2 and 3) or key individual crop yields (Fig. 4) following paraquat bans. On the contrary, in several countries, yield increased following the ban, e.g., Senegal and Mauritania. We acknowledge that there are several other factors not considered in this analysis that affect crop yields, such as weather effects, pest or disease outbreaks, policy shifts, and/or large-scale changes in farm practice. However, the data indicates that crop productivity at a national level has not been negatively affected by paraquat bans in all cases analyzed. In the next section, we identify the alternative methods for weed management that are available and highlight the growing body of literature that suggests substantial reductions in overall herbicide use could be achieved without negatively affecting agricultural productivity.

## Alternative methods for weed management

There are a wide range of non-herbicide and herbicide—both synthetic and plant-derived—tools and methods available to farmers that can be used as a direct alternative to paraquat or as part of an integrated weed management (IWM) system to replace its use (PAN Europe 2018, PAN Germany 2008, Riemens et al. 2022, Singh et al. 2014). Agroecological approaches can also considerably reduce the need to kill plants with either herbicides or mechanical techniques, with improved environmental outcomes (Gliessman 2014; Liebman et al. 2001). The following section describes these methods.

### Integrated weed management

The key to reducing or eliminating herbicide use is to integrate a variety of physical, cultural, and ecological methods, as part of an IWM approach. The aim of IWM is to diversify weed management strategies and reduce reliance on herbicides (Moss 2019). IWM reduces the negative effects of herbicides on humans and the environment, including on soil health and agronomic sustainability. Repeated use of any weed management method, such as repeated paraquat use, provides heavy selection pressure for weed adaptation and resistance; IWM minimizes this problem (Harker and O'Donovan 2017) by making use of a “*many little hammers”* (i.e., multiple weed management tools) approach as opposed to reliance on one single method. IWM is increasingly recommended for dealing with herbicide-resistant weeds, phasing out specific herbicides or reducing overall reliance on herbicides (Grow IWM 2022, PAN Europe 2018; Peachey 2022).

Following a systematic review of the literature, Colbach et al. (2020) concluded that reducing herbicide use rarely results in reduced crop yields due to weeds if farmers use other efficient agricultural practices which make the field a less favorable environment for weeds (often referred to as “cultural, cultivational, or weed preventative practices”). Their review highlighted many field studies that show that IWM can be highly efficient in the management of herbicide-resistant weed populations, thus minimizing the need to use more herbicides. Riemens et al. (2022) take this further by arguing for a paradigm shift in weed management, from a focus on herbicide efficiency and substitution to the redesign of cropping systems. The authors suggest that, instead of aiming for weed eradication, the goal should be to reduce the negative effects of weeds, while retaining some ecological benefits. For example, many plants considered to be weeds may provide important habitat or food for pollinators and other beneficial insects, provide an important food source for rural communities, help to suppress problematic weeds, or provide valuable ground cover and protect the soil (and soil microorganisms) from sun/rain damage and erosion (Adeux et al. 2019, Brühl and Zaller 2021, Daum et al. 2022, Lampkin 1990, Neumeister 2016, Sánchez-Bayo 2021).

Reflecting these changing attitudes, Merfield (2022) created a new definition for weeds. He defines a weed as “a plant, or population of plants, in a specific time and place, causing significant harm, either immediately or in the longer term, based on a holistic analysis of both their positive and negative attributes.” This moves weeds from the common definition of plants that are “not wanted” to plants causing “significant harm,” thus considerably raising the bar that defines a plant as a weed. This aligns with the promotion of an agroecological approach as an integral part of successful IWM (Tataridas et al. 2022). Diversifying cropping systems and weed communities and supporting ecosystem services ensure the functionality and the sustainability of agricultural systems which have the potential to provide economic and ecological benefits in both the short- and long-term (Adeux et al. 2019; Chandrasena 2019; Gaba et al. 2016; Low 2020; Rosa-Schleich et al. 2019; Sharma et al. 2021).

### Herbicide alternatives to paraquat

There are numerous available herbicides that are used as a direct substitute for paraquat (Beckie et al. 2020; Kim and Kim 2019; Marambe and Herath 2019, Neumeister 2016). These most commonly include diquat, glufosinate ammonium, and glyphosate. However, all synthetic chemical herbicides can have a range of adverse health and environmental effects that need to be taken into consideration. For example, following the EU ban on paraquat, diquat, another bipyridylium herbicide with a similar mode of action, was used as a substitute for paraquat in the UK for pre-harvest potato haulm dessication. However, due to its high mammalian toxicity, diquat was later banned by the EU (EC 2018).

Neumeister (2016) argues that numerous herbicides should not be considered as alternatives to paraquat because of their high mammalian toxicity or potential for chronic health or environmental effects, as estimated using the Toxic Load Indicator (TLI) methodology (Neumeister 2017). Table 2 summarizes the toxic load scores for paraquat and nine other commonly used herbicides using criteria selected for different aspects of mammalian acute toxicity and long-term health effects, for ecotoxicity to key groups of plants and animals, especially those providing ecosystem services, and for environmental fate and mobility. Many herbicides have a low acceptable daily intake (ADI) or acceptable operator exposure level (AOEL), indicating that repeated exposure in excess of those levels could pose substantial health risks. Paraquat, diquat, dicamba, diuron, glufosinate ammonium, and haloxyfop-R-methyl all fall into that group. 2,4-D, diuron, and glyphosate are of concern in relation to carcinogenicity and glufosinate ammonium due to reprotoxicity. Besides health concerns, many common herbicides also pose significant risks to beneficial organisms and the environment. However, the TLI indicates that some herbicides are considerably less toxic than paraquat, e.g., imazapyr and carfentrazone-ethyl.

**Table 2:** Toxic Load Indicator (TLI) values for 12 herbicides used as alternatives to paraquat (source: adapted from Neumeister (2016) and updated by L. Neumeister, May 2022)

**#**				Mammalian toxicity (MT)						Environmental toxicity (ET)						Environmental fate and transport				
	Active ingredient	Total TLI score	Sum 1 MT WF^a^ = 2	Acute toxicity	Carcinogenicity	Repro. Tox	Mutagenicity	AOEL^b^ /ADI^c^	Sum 2 ET	Algae	Daphnia /fish	Birds	Bees	Beneficials	Sum 3 EF and T	Bioaccumulation	Persistence	Half-life on plant	Leaching potential	Volatility
1	Imazapyr	**35**^d^	***12***^e^	1	1	1	1	2	***10***	1	2	1	1	*5*	***13***	1	1	1	5	5
2	Carfentrazone-ethyl	**43**	***24***	1	1	1	1	8	***13***	5	5	1	1	*1*	***6***	1	1	1	1	2
3	Dicamba	**65**	***34***	5	5	1	1	5	***12***	5	2	2	1	2	***19***	1	8	1	1	8
4	Imazethapyr	**68**	***34***	1	1	5	5	5	***9***	1	1	1	1	5	***25***	1	8	1	10	5
5	Glyphosate	**69**	***38***	2	10	1	1	5	***14***	5	2	1	1	5	***17***	1	5	5	1	5
6	2,4-D	**74**	***46***	5	8	1	1	8	***14***	5	5	2	1	1	***14***	1	1	5	5	2
7	Haloxyfop- R-methyl	**75**	***30***	2	1	1	1	10	***28***	5	10	2	1	10	***17***	1	1	5	*5*	5
8	Glufosinate ammonium	**81**	***54***	5	1	1	10	10	***14***	1	1	1	1	10	***13***	1	1	5	1	5
9	Paraquat dichloride	**88**	***42***	8	1	1	1	10	***24***	5	5	8	1	*5*	***22***	1	10	5	1	5
10	Diquat dibromide	**90**	***42***	8	1	1	1	10	***26***	5	5	5	1	10	***22***	1	10	5	1	5
11	Diuron	**90**	***48***	2	10	1	1	10	***19***	10	5	2	1	1	***23***	1	10	5	5	2
12	Quizalofop-P-ethyl	**91**	***54***	2	5	5	5	10	***23***	5	8	1	1	*8*	***14***	5	1	5	1	2

^a^*WF*, a weight factor of 2 is given to human health toxicity, i.e., the toxic load sum is doubled compared with environmental toxicity, fate, and transport to reflect the priority concerns to protect human health, and a default value of 5 is given for any criteria where no data is available for a particular active ingredient

^b^*AOEL*, acceptable operator exposure level

^c^*ADI*, acceptable daily intake

^d^Numbers in bold signify the total TLI score

^e^Numbers in italic signify the sum score per TLI category

A number of natural herbicides are available for weed control (Ciriminna et al. 2019; Cordeau et al. 2016; Flamini 2012). Experience with alternative products based on botanical extracts is growing, although for those with direct contact, burn-down action care must be taken to avoid contact with the crop, e.g., with use of protective spray shields on the sprayer equipment (Osuch et al. 2020). In Latin America, one product, Sec Natural, based on conifer and other botanical oils can work well without spray shields in crops with thick, waxy cuticles, e.g., citrus and pineapple (Perez, R, pers.comm, Costa Rican Association for the Study of Weeds, i.e. ACEM). Another botanical product, Herbor G, registered as an organic herbicide gave results as good as paraquat for foliage burn-down pre-planting in Costa Rican organic sugarcane trials, while proving less costly than manual weeding (Arias, O., pers.comm, ACEM).

### Non-herbicide alternatives to paraquat

There are a diverse range of non-herbicide weed management approaches which can serve as effective replacements for paraquat and other herbicides. These range from traditional techniques such as inter-row hoeing, to modern adaptions using computer vision systems. Novel “high-tech” approaches such as fully autonomous robotic weeders operating at the individual plant level could facilitate truly selective weed removal—something that is impossible with any herbicides using standard “broad acre” spraying. Before herbicides were developed, mechanical weeding using harrows, inter-row cultivators or mowers were widely used in farming and remain the most common alternative to herbicide use for direct weed control. They are successfully used in organic and non-organic farming (Riemens et al. 2022; Zaller 2020).

In addition to the use of mechanical methods, other agroecological practices, such as mulches, cover crops, livestock grazing, crop rotation and diversifying crops and weed communities, provide a holistic and more sustainable approach to managing weeds. These practices provide multiple ecosystem services, as well as improving profitability due to reduced costs and increased crop yield (Adeux et al. 2019; Crézé and Horwath 2021; Hartwig and Ammon 2002; Tataridas et al. 2022). Over 100 beneficial microorganisms have been identified as potential weed biocontrol agents (Harding and Raizada 2015). Most of these are fungi, but bacteria and viruses are also attracting attention and about a dozen bioherbicide products, based on strains of specific fungi or bacteria, are available commercially (Triolet et al. 2020). The Centre for Agriculture and Bioscience International (CABI) recently launched a Bioprotection Portal (https://bioprotectionportal.com) which provides information on national registrations for these products for over 30 countries.

The published literature on non-herbicide alternatives specifically in relation to paraquat is limited, mainly because most non-herbicide weed management research does not aim to directly replace specific herbicides; rather, they aim to create integrated non-herbicide weed management techniques and systems. However, a large amount of research has been conducted on non-herbicide methods of weed control and IWM in general that are applicable for replacing paraquat use. Non-herbicide methods of weed control have been summarized in many reviews (Colbach et al. 2020; Harker and O'Donovan 2017; Korres et al. 2019a; MacLaren et al. 2020; Melander et al. 2017; Merfield 2019; Moss 2019; Tataridas et al. 2022). We provide some examples of direct alternatives to paraquat below, along with further detailed case studies in subsequent sections.

#### Living mulches

Living mulches (also known as cover crops or service plants) are low growing perennial plant species grown underneath perennial crops such as trees and vines, for weed management as well as for nitrogen fixation, provision of resources for beneficial species that control arthropod pests, reduced soil erosion and run off, soil health, etc. Living mulches are increasingly used in organic tree and vine crops as well as by non-organic producers aiming to reduce herbicide use, soil erosion, and pesticide run-off (Prosper et al. 2019; Staver et al. 2020a; Tardy et al. 2017). The living mulch may be spontaneous natural vegetation, with some selective weeding to favor low-growing, mat-forming, non-competitive species, or deliberately sown, often a legume species for nitrogen fixation. Living mulches are highly beneficial on sloping fields where mechanical weeding is impractical and erosion may be a problem; the plant foliage protects soil from the effect of rain and impedes overland flow, and the roots bind the soil, all of which significantly reduce water and wind erosion. Living mulches are also being tried by farmers in temperate zones for cereal rotations (e.g., https://www.innovativefarmers.org/case-studies/managing-living-mulches-on-arable-farms/).

Trials in the early 2000s in the Windward Islands (Lesser Antilles, Caribbean) to find alternatives to herbicide use compared “living mulches” of wild peanuts (*Arachis pintoi*), velvet beans (*Mucuna pruriens)*, and the tropical forage legume *Desmodium heterocarpon* with various herbicide and mechanical treatments. *D. heterocarpon* worked the best as a living mulch in weed suppression and performed better than some herbicides and mechanical treatments. In follow-up trials, farmers and researchers agreed that *D. heterocarpon* could significantly reduce weed levels and provide a sustainable non-herbicide approach to improve weed management of the key problem weed, climbing dayflower (*Commelina diffusa*), in banana fields (Isaac et al. 2007, 2012). The use of paraquat for the control of parasitic witchweed (Striga spp.) in several tropical crops can be substituted by planting allelopathic plants, including crops that have relatively high resistance to witchweed (Samejima and Sugimoto 2018) and companion plants, such as *Desmodium* spp. that reduce parasitism rates (Khan et al. 2002). The use of *Desmodium* to control witchweed is increasingly adopted by farmers in Africa (Midega et al. 2017).

#### Controlled grazing

Grazing livestock to control grassy and broadleaved weeds is used in coffee, oil palm, banana and orchard crops, and in vineyards, by both organic growers and non-organic growers. In Malaysia, integrating cattle grazing into oil palm plantations to reduce paraquat and other herbicide use reduced labor costs by up to 50% and herbicide costs by 30–50%, while increasing oil palm fresh bunch yields by 6–30% and improving avian biodiversity and soil structure through the addition of organic matter to the soil (Lam et al. 2009; Tohiran et al. 2017). Small-medium scale organic banana growers in the Dominican Republic make use of chickens, up to 30 hens per hectare, grazing from early morning until afternoon, which only occasionally require monitoring. Alternatively, sheep or goats may be used, as long as there is some broad-leaved vegetation present; otherwise, goats may browse the young banana suckers (Gandini 2021). In New Zealand, large flocks of sheep are commonly used in vineyards after harvest, resulting in 1.3 fewer herbicide applications annually on average and saving US$56/ha (Niles et al. 2017).

#### Mechanical weeding

Mechanical weeding covers an exceptionally wide range of techniques for both crop production and urban weed management. Prior to the advent of the synthetic herbicides from the 1940s onwards, the default form of weed management in agriculture was mechanical (Timmons 2005). Modern mechanical techniques have built on that foundation and produced a wide range of novel approaches that have dramatically broadened the techniques used to kill weeds, increasing their effectiveness and speed, especially with the use of computer vision and global positioning guidance systems (Hussain et al. 2018; Merfield 2018).

Mechanical weeding begins with cultivation/tillage techniques such as ploughing, and creating false and stale seedbeds, which encourage weed seed germination so that these can then be controlled before the crop is sown (Merfield 2015). Paraquat is widely used for stale seedbeds, but machines such as false seedbed tillers provide equally effective control of small weeds pre-planting (Merfield 2018). Following crop emergence, paraquat use can be replaced by a huge selection of inter-row hoes that weed between the crop rows. There are also a wide and increasing range of intra-row weeding tools that can control weeds in the crop row, a job that paraquat and other non-selective herbicides cannot do (Hussain et al. 2018; Merfield 2018).

There are legitimate environmental concerns about soil damage from repeated mechanical weeding and soil loss to wind and water erosion, with moves in many farming systems to reduce tillage to protect soils. However, modern equipment and combining partial mechanical controls with other IWM methods can avoid serious damage, while conventional arable farmers may benefit from mechanical techniques used by organic farmers (Alford 2018). Reduced and no-till conservation agriculture is thus possible without the use of herbicides (FAO 2020, Watts and Williamson 2015).

In recent years, robotic weeders have made considerable progress (Bawden et al. 2017; Korres et al. 2019b). These identify individual crop and weed plants and selectively kill the weed plants using a wide range of tools including mechanical (e.g. small hoe), electrothermal, hot oil, focused light, lasers, and micro-doses of herbicides (Bawden et al. 2017; Fennimore and Cutulle 2019; Young et al. 2017). The logical endpoint of this approach allows for truly agroecological weed management by deciding which individual non-crop plant species are non-harmful, or even beneficial, based on their species and populations and leave them to grow with the crop, only killing individual weed plants that will negatively effect the crop (Adeux et al. 2019; Gerowitt et al. 2003; Storkey and Neve 2018).

#### Thermal weeding

Thermal weeding provides a diverse range of options that can be direct replacements for paraquat. Thermal weeding is defined as any weeding technique that uses heat or cold to kill weed plants. It has a similar “mode of action” to paraquat, as indicated by the term “burn down” to describe paraquat’s effect on plants. All commercially available thermal weeding systems use energy to boil the water inside the plant causing destruction of the plants cellular structure. The effects are rapid, from seconds to hours and are visually obvious, e.g. plants wilt and/or turn brown (Bond et al. 2007, Peerzada and Chauhan 2018).

Despite considerable research and development, only a small subset of thermal weeders are sufficiently effective, economic, practical, and safe to be in widespread use. Flame weeding is by far the most common and low-cost approach, which uses open flames typically produced from petroleum gasses, e.g. propane (Datta et al. 2013). Flame weeding in agriculture is most widely used in vegetable and row-crop production against small annual weeds, and, to a lesser extent in urban areas for weed control on hard surfaces (Hansson 2002). Paraquat can be directly substituted by flame weeding in a range of situations where smaller weeds need to be killed or larger weeds defoliated.

Steam is the next most widely used thermal method, although the approach is still comparatively uncommon. It has the same effect on weeds as flame weeding. However, steam does not have the fire risk associated with flame weeding, thus is preferable in perennial crops and urban areas, where fire is a risk (Merfield et al. 2009). Steam can be used as a direct replacement for paraquat in a range of production and urban situations.

Electrothermal weeding uses high voltage (1,000 to 15,000 kV) to directly boil the water inside weeds, and/or disrupt cellular function and structure (Diprose and Benson 1984; Diprose et al. 1985; Eberius 2017; Merfield 2016). It is unique among thermal weeding approaches as it is partly systemic, as the electricity travels through the plant foliage, through the hypocotyl, and into the top of the root system before exiting into the soil (Diprose and Benson 1984; Merfield 2016). By using different application methods, electrothermal weeding can be used in many different ways to directly replace paraquat and other herbicides in agricultural and urban environments. Electrothermal equipment is starting to be used in Latin America for inter-row weeding in broad-acre crops in organic systems and as a good tactic to deal with herbicide-resistant weeds in conventional systems. According to the distributor, the technique offers major advantages in reducing the number of passes needed compared with purely mechanical weeding and claims no significant negative effects on soil microbes or insects (Garnham, E., pers. comm). One sugar cane producer described zero herbicide residue as a major benefit of electrothermal weeding in marketing terms, even if the duration of control was not as long as with herbicides, as well as the fact that it worked well on hard to control *Cyperus* spp. (Mejia, M., pers. comm.). Applied to a whole field surface, it replaces glyphosate and paraquat in no-till systems. Electrothermal, flame and steam are also used for crop desiccation, e.g. for the destruction of haulm on potatoes (Knezevic et al. 2014; Meyer 2021).

## Experiences from private voluntary standards (PVS) and supply chain actors

Numerous PVS have evolved since the 1990s to address food safety, food quality, and environmental and social effects and to aim for more sustainable agri-food value chains (Djama 2011, Gereffi et al. 2005; Henson and Humphrey 2010). There are diverse opinions about their effect (Dietz and Grabs 2022; Mengistie et al. 2017; Raynolds 2012; Schreinemachers et al. 2012). This section gives a short overview of the status of paraquat in selected PVS before outlining successes and challenges in paraquat phase-out in six crops in different PVS.

No comprehensive database exists comparing how paraquat is addressed by the plethora of different PVS or individual retailers. However, the ISEAL Integrated Pest Management (IPM) Coalition’s on-line database (https://www.ipm-coalition.org/) allows users to search and compare requirements on paraquat and hundreds of other HHPs among eleven ISEAL members. Table 3 provides a summary of current status of paraquat among six ISEAL member standards, which cover cotton, sugarcane, forestry, coffee, and other tropical export crops, plus two certification schemes on soya and palm oil. Three standards, Rainforest Alliance, Fairtrade, and Forest Stewardship Council (FSC), have long prohibited paraquat among their producers. Better Cotton Initiative (BCI) has a phase-out deadline of 2024, although several BCI producer countries have already achieved phase out (Jean, G., pers. comm.). One standard (Bonsucro) has no indicated paraquat restrictions, while the extent of paraquat use restrictions are unclear for Roundtable on Sustainable Palm Oil (RSPO) and Roundtable on Responsible Soy (RTRS).

**Table 3:** How paraquat is currently handled by selected private voluntary standards (PVS) (see Supplementary file for the list of information sources)

PVS	Status of paraquat use
Better Cotton Initiative (BCI)	Planned phaseout by 2024^a^
Bonsucro (sugar)	Unrestricted
Fairtrade International (various crops)	Full prohibition since at least 2005
Forest Stewardship Council (FSC)	Prohibition since at least 2005
Global Coffee Platform (GCP)	Red Listed, with expected max. phase out period of 3 years from each producer’s date of joining
Rainforest Alliance (various crops)	Full prohibition since 1993, starting with banana
Roundtable on Sustainable Palm Oil (RSPO)	Status unclear^b^
Roundtable on Responsible Soy (RTRS)	Status unclear^c^

^a^Not currently restricted under BCI but would become prohibited if specific paraquat severely hazardous pesticide formulations become Rotterdam-listed, as recommended by Rotterdam Convention Chemical Review Committee

^b^RSPO standard says that “Pesticides that are categorised as World Health Organisation Class 1A or 1B, or that are listed by the Stockholm or Rotterdam Conventions, and paraquat, are not used, unless in exceptional circumstances, as validated by a due diligence process, or when authorised by government authorities for pest outbreaks”

^c^RTRS standard says that “There is no use of agrochemicals listed in the Stockholm and Rotterdam Conventions” and that “*Paraquat and Carbofuran are banned according to the Stockholm and Rotterdam Conventions*.” However, this is incorrect as paraquat is not yet listed on the Rotterdam PIC list

### Replacing paraquat use in soybean production

Herbicides, notably paraquat and glyphosate, has been an issue of human health concern in small and large-scale soybean cultivation, especially in South America (Gross 2018, Phélinas and Choumert 2017). RTRS is one PVS aiming to improve sustainability in the conventional sector. As part of a stakeholder consultation in 2015–2016, RTRS sought feedback on whether to follow the lead of other PVS, such as Fairtrade and Rainforest Alliance, and introduce a prohibition or restrictions on paraquat use in soybean supply chains. In 2016, World Wide Fund for Nature (WWF) Germany also commissioned a report on alternatives to paraquat for use on soybean in Argentina, Brazil, India, and Uruguay (Neumeister 2016). It identified 38 alternative non-HHP herbicide active ingredients (at least eight registered in each country), presenting lower human and environmental risks than paraquat. In India, there were no approved uses of paraquat in soybean, while research showed that best weed control results in this crop were delivered by combining a low dose of diclosulam with one round of hand weeding (Nainwal et al. 2010).

In May 2016, on the basis of their technical assessment, WWF Germany and others, including two Swiss retailers, strongly urged RTRS to completely prohibit paraquat by 2017, advocating for an integrated approach with crop rotation, manual control, and less toxic herbicides effective at low dose. They noted that several RTRS certified producers were already producing soy without using paraquat and any continued use of paraquat would hamper the uptake of RTRS in Europe by creating reputational risks for European companies. So far, it is unclear to what extent paraquat has, or has not, been phased out in RTRS soy production, although the start of Brazil’s national paraquat ban implementation in 2020 should trigger a rapid change to alternatives in soy production country-wide and strengthen arguments for RTRS to push for a definitive prohibition across all its soy producing members globally.

### Replacing paraquat use in oil palm

Oil palm plantations have long been the target of civil society campaigns in Southeast Asia and beyond to prohibit use of paraquat, due to high reported levels of risky handling practices and of occupational poisonings (Kamsia et al. 2014; Myzabella et al. 2019; PANAP 2017, Tenaganita and PANAP 2002). In 2011, CABI was commissioned to assess herbicide use in oil palm production and identify potential alternatives (Rutherford et al. 2011). Researchers surveyed weed management practices by selected producers in Malaysia, Indonesia, and Papua New Guinea to collate information on herbicide and non-herbicide methods used for ground cover management, as well as their cost-effectiveness as perceived by the producers.

Fifteen synthetic chemical herbicides were used by survey respondents. Glyphosate and metsulfuron were used by almost all, and 2,4-D, triclopyr, and paraquat were each used by roughly half of the respondents. All responding producers regularly monitored ground cover vegetation on their plantations and all managed growth by planting a cover crop(s) and through application of herbicide. Most applied organic mulch, while many relied on hand pulling, slashing, or the use of a hoe (see Table 4).

**Table 4:** Weed management methods used by oil palm producers surveyed in Indonesia, Malaysia, and Papua New Guinea (source: adapted from Table 1 in Rutherford et al. (2011))

Ground cover management method	% producers using in mature oil palm crop (*n* = 25)
Herbicide application	96
Organic mulch	88
Slashing	56
Uprooting plants with a hoe	56
Cover crop planted	40
Mechanical (mower, tractor)	36
Uprooting plants by hand	32
Biological control	32
Grazing by livestock	28
Plastic sheeting mulch	4
Increased palm planting density	4

The use of herbicides, cover crops, and mulch by producers was reflected by their perceived cost-effectiveness in comparison with other methods. In contrast, manual approaches to weeding were considered to be much less cost-effective. Several other IWM methods, however, including mechanical weeding, increasing palm density, covering the ground with sheeting, and grazing by livestock, were considered to be more cost-effective, yet used by few producers. The authors of the report commented that these non-herbicide methods might have potential for broader uptake by producers, especially if underlying reasons for the observed poor rate of adoption could be understood and addressed.

Case studies from four producers in the same focus countries showed that elimination of paraquat use was achieved partly by replacing it with less hazardous products and/or partly by adoption of non-herbicide approaches—specifically, manual and mechanical weed management, application of various mulches, and cultivation of cover crops. The authors highlighted that, in many instances, IWM methods were considered to not only be safer but more efficient and more cost-effective than using herbicides.

The CABI assessment concluded that a range of herbicide and non-herbicide measures were available for effective weed management in oil palm, and that reductions in herbicide use were readily achievable through a variety of non-herbicide methods and also more rational use of herbicide products and/or paraquat substitution with less toxic substances. They emphasised how, to be successful, measures must be adopted within an integrated approach to weed control, as opposed to being used in isolation.

Data from one recent Indonesian study on effectiveness of certified standards in improving environmental performance among smallholder oil palm growers suggested that RSPO certified smallholders were much less likely to use paraquat: 0–11% reported use in two types of RSPO smallholder groups, compared with 34–95% of smallholder groups certified by the national oil palm standard and with 30% of uncertified growers (Chalil and Barus 2020).

More recent research from one Indonesian plantation confirms the economic and ecological viability of less intensive management practices and replacing herbicide use with mechanical weeding (Darras et al. 2019). Plants, above-ground arthropods, and below-ground fauna were positively affected by mechanical versus chemical weed control, while no detectable negative effects of reduced fertilizer use or mechanical weeding were found on oil palm yields, soil nutrients, and functions (mineral nitrogen, bulk density, and litter decomposition). Water infiltration and base saturation also tended to be higher under mechanical weeding.

### Replacing paraquat use in coffee

Fairtrade and Rainforest Alliance certified coffee farmers have been prohibited from using paraquat for at least 15 years (Table 3). This prohibition currently applies to around 1.23 million small and medium-scale coffee farmers—almost 10% of the estimated 12.5 million coffee farms in this category (WCR 2021)—and numerous large estates in Latin America, Africa, and Asia (Fairtrade 2020, Rainforest Alliance 2021). These producers are successfully growing and selling high-quality coffee; several initiatives are supporting them to reduce reliance on herbicides or even eliminate them. Most focus on implementing IWM approaches, often making use of ground cover vegetation, either leaving naturally occurring non-competitive plants to thrive or sowing leguminous cover crops, with considerable co-benefits for soil conservation, moisture retention, and biodiversity, particularly for, as well as avoiding economically damaging weed problems (CATIE and Rainforest Alliance 2021, PAN UK 2016b, Ramírez 2021). Many coffee farmers have learnt to combine ground cover with some level of mechanical/manual weed control, mulching with prunings from shade trees and weed cuttings and grazing by small livestock. This has provided soil health benefits and reduced fertilizer application rates (Bellamy 2011, Gamboa and Umana 2018, Staver et al. 2020b).

From the mid-1970’s, glyphosate and several other systemic herbicides, e.g., glufosinate ammonium, became available and partially replaced paraquat. However, these alternatives are not without their problems. Several authors have documented phytoxicity issues and highlighted the harmful effects of reliance on high-dose, blanket applications of glyphosate on young coffee trees (Castanheira et al. 2019, Chaverria 2018, Nelson 2008). Phytotoxic damage from drift of glyphosate droplets in coffee seedlings is common, and, in addition, there are reports of contamination by this herbicide to non-target plants via the rhizosphere (Barbosa et al. 2020). In addition, Costa Rica’s Regional Institute for Research on Toxic Substances (IRET) has documented increasing problems of glyphosate phytotoxicity and how this is triggering non-certified farmers to return to spraying paraquat (Ramírez 2021). Paraquat import volumes into Costa Rica remain high, at over 870,000 kg active ingredient per annum and the third most imported pesticide (by volume) after mancozeb and glyphosate (Vargas 2022). This is of concern to IRET and other members of the pesticides working group of the National Secretariat for Chemicals Management.

In efforts to replace both paraquat and glyphosate use in coffee within Costa Rica, there is growing interest in the introduction of botanical herbicides, which are used elsewhere in Latin America, e.g., Mexico. However, registrations for their use in Costa Rica are still at the initial stage. In general, there is increased awareness of the negative effects that herbicides can cause in coffee production systems, especially by small and medium scale farmers in hillside areas. This awareness has led to a reduction in herbicide use and increased acceptance of the concept that weeds are a necessary component of coffee agroecosystem.

### Replacing paraquat use in tea

There are over 200,000 ha of tea in Sri Lanka, of which almost 60% is in smallholdings. These smallholdings are highly productive and produce 73% of Sri Lanka’s tea (compared to 20% from large plantations). Weed management has always been a major cost for Sri Lankan tea production, second only to harvesting, with an estimated 800% increase since the 1990s. The use of herbicides, including paraquat, has been the main method for managing weeds in almost all large tea plantations, although less than 10% of tea smallholdings currently use chemical herbicides (ASLM 2022). Reliance on herbicides in large plantations resulted in the rapid development of resistant weed populations, for example, of the 23 most damaging weeds in Sri Lankan tea, 20 had resistance to both paraquat and glyphosate (Peiris and Nissanka 2016).

Following a national paraquat ban in 2012 and restrictions on the use of glyphosate in Sri Lanka in 2014 (Marambe and Herath 2019), alternatives to herbicide use in tea were sought, with support from the Global Environment Facility to set up Herbicide-Free Integrated Weed Management (HFIWM) validation sites and to promote this technology among farmers. Herbicide-free techniques, already applied in India for tea with promising results, involve the manipulation of ground-cover plants through the selective removal of noxious weeds and the promotion of beneficial flora. The method involves training of tea workers to distinguish damaging (so-called “hard”) plants from beneficial or non-damaging (“soft” or “innocent”) ones; “hard” weeds are removed before they seed, and “soft” weeds are allowed to seed, after which they are manually removed. Over time, the tea plantation or smallholding becomes dominated by “soft” weeds. This technique has been monitored for cost-benefits and found to not only reduce fertilizer and labor costs but to also increase yields (possibly by reducing the phytotoxic effects of chemical herbicides on the tea plant). HFIWM advantages include gains from mulching and composting, as well as achieving several other sustainability indicators (Gunarathne and Peiris 2017; Peiris 2016). Facilitated through support from both the public and private sector, including the Rainforest Alliance, the uptake of herbicide-free weed management is growing in Sri Lanka, already with an estimated 15,000 farmers trained in the technology (ASLM 2022).

### Replacing paraquat in potato haulm desiccation

Paraquat and diquat have been the predominant herbicides used in haulm desiccation prior to potato harvesting to aid the harvest process (Da Silva et al. 2021; Griffin et al. 2022). Paraquat has been reported to have a low effect on potato quality (Da Silva et al. 2021); however, the risk and possible effects of residues remaining in harvested tubers are likely affected by environmental conditions, including weather (Krupek et al. 2021). Paraquat was preferred because it is fast acting, compared to other herbicides (Da Silva et al. 2021). Together, these factors raised the risks of an overuse of paraquat during haulm desiccation, with potential consequences for human and environmental health.

The ban of both paraquat and diquat in Europe has led to increased investments in the search for suitable alternatives. Among the most popular alternatives is mechanized flailing, sometimes followed by flaming or a lower toxicity herbicide (Kardasz et al. 2019). Mechanization of haulm removal has required changing planting times in parts of northern Europe to avoid the operation of heavy machinery during times when the soil is soft due to rain (Griffin et al. 2022; Krupek et al. 2021). Despite the ban of the predominant herbicides used for potato haulm desiccation, yields per hectare have consistently increased in countries like Ireland, where soil saturation can limit the operation of heavy machinery prior to late-season potato harvests (Griffin et al. 2022). The development of lighter, more effective machinery will likely capitalize on the paraquat and diquat bans to improve the sustainability of potato production in northern Europe. For example, the Irish Government has set alternatives to herbicide use in haulm desiccation as an objective in their Targeted Agricultural Modernization Scheme (DAFM 2022).

### Replacing paraquat for pineapple foliage destruction

One specific and relatively recent use of paraquat involves the need to achieve rapid rotting of pineapple foliage post-harvest in Costa Rica. Pineapple cultivation produces about 250 tons of crop waste per hectare (Lopez-Herrera et al. 2014), which needs to be treated quickly or removed from the field to prevent the spread of the stable fly *Stomoxys calcitrans*, a livestock pest. Rotting pineapple foliage is highly attractive to stable fly for egg-laying and if poorly managed can become a breeding ground for this economically harmful pest (PAN UK 2016a). Until 2005, it was common practice for most pineapple producers in Costa Rica to avoid this problem by desiccating foliage with very high volumes of paraquat, followed by burning. This practice, however, led to serious environmental contamination and problems of soil erosion. Numerous pineapple supply chains have been working to manage pineapple foliage waste and stable fly populations without resorting to paraquat (PAN UK 2017, UNEP 2012).

Rainforest Alliance has prohibited paraquat since 1993, with the creation of the sustainable agriculture standard, applicable first for bananas only, but opened to other crops a few years later. Stable fly is a particular challenge to the 45 Costa Rican pineapple producers growing to the Rainforest standard. In response, the standard identified a series of good agricultural practices, such as stubble management, and preventing, monitoring, and sustainably controlling different pests. By 2020, 95% of Rainforest certified pineapple producers had received relevant training, and none were using paraquat (M-A Bonilla, Rainforest Alliance, pers. comm). Most producers now reincorporate pineapple organic matter into the soil (a valuable nutrient and soil structure improver) and some apply decomposer microbes to the post-harvest foliage (UNEP 2012). This greatly reduces the time that the crop waste can serve as an attractive breeding site for stable fly.

While there are sometimes constraints with availability and quality of the microbial products, uptake of this agroecological solution to pineapple waste has been rapid and widespread among most Rainforest Alliance producers. Rainforest Alliance reports there is no yield penalty, and the practice delivers considerable benefits for soil health in the medium term from returning 250t/ha organic matter to fields. This improves soil nutrients, structure, and acidity to levels that are optimum for pineapple growth and produces healthier plants which are better able to resist pests and/or disease attack.

Since 2018, the pineapple company Nicoverde has promoted practices to reduce reliance on pesticides and boost on-farm biodiversity, trained producers, and run demonstration plots. They have documented four seasons of success in dealing with post-harvest crop waste without paraquat on their own 110 ha pineapple farm and from 100 small- and medium-scale pineapple growers with a mean farm size of 4.4 ha. Their field-validated protocol consists of mechanical chopping/harrowing, followed by four applications of a tailor-made selection of decomposer microbes, produced in Nicoverde’s own biolab and sold at 50% discount to Nicoverde growers. About 3–4 weeks after treatment, the decomposing material is then incorporated into the soil. This practice is effective in preventing build-up of stable fly populations and contributes to regenerating soil health and protecting groundwater from pollution. Growers expressed satisfaction with the crop waste management protocol because it avoids the health risks from paraquat spraying, while improving soil quality.

Careful planning and timing of foliage treatment or destruction and subsequent replanting is an important consideration in achieving good control of stable fly without resorting to paraquat, especially in zones with heavy rain periods, when tractor-mounted machinery cannot be used.

## Lessons learnt from public and private sector bodies

### Lessons learnt from the perspective of supply chains

Numerous supply chains, both those involving PVS and those growing for specific retailer companies, have taken the initiative to phase out paraquat and other HHPs ahead of any national bans or restrictions. We sent an adapted survey questionnaire in December 2021 to five ISEAL member organizations to find out their experiences in paraquat phase out. A similar questionnaire was also sent to the top ten UK retailers.

Two ISEAL members and four major UK retailers responded. Table 5 summarizes the current status of paraquat use among the four UK retailer respondents. For three of these four retailers, the only permitted remaining use is for the highly specific situation of pineapple foliage removal as a breeding site for stable fly. The fourth also identified limited potential remaining uses for weed control in avocado in Colombia and kiwi fruit in Chile, although not necessarily used in practice. Two retailers indicated a major reduction in paraquat use and change in practices by their Costa Rican pineapple growers in recent years, as a result of joint actions to address this issue. Numerous suppliers now implement a similar system for mechanical shredding and microbial degradation as featured in the pineapple case study in the “Replacing paraquat for pineapple foliage destruction*”* section. One major importer supplying several retailers noted that the land preparation time using the newest shredding equipment is six weeks shorter than when using paraquat as desiccant, while time using the standard shredders is the same as for paraquat use. This is an economically significant consideration for larger pineapple producers in particular, as their retailer customers demand continuous production of large volumes year-round. These responses demonstrate the feasibility of phasing out paraquat in pineapple, particularly when growers and retailers work together to this end.

**Table 5:** Status of paraquat use among four UK retailers. Responses from technical managers of four UK retailers to lead authors’ questionnaire survey (conducted between December 2021 and February 2022)

Response topic	Retailer A	Retailer B	Retailer C	Retailer D
**Restrictions on paraquat use**				
Paraquat use to be phased out as a priority			□	
Paraquat use restricted-only permitted by BASIS^a^ qualified agronomist, for one year only, and contingent on a specific IPM-based justification for use	□			
Paraquat use virtually eliminated, except in rare, single farm particular cases		□		
Paraquat on company Prohibited List and no use allowed anywhere in company				□
**Extra requirements when use is approved**				
Any use approvals require extra mitigation measures, operator training and demonstration of a robust management system	□	□	□	n/a
Growers using paraquat must also report on progress to develop/deploy alternatives for foliage destruction	□		□	
**Status of IPM alternatives for pineapple foliage treatment**				
Actively working with Costa Rican suppliers to implement alternatives	□	□	□	□
Implementing mechanical shredding + microbial degradation	No info	□	□	□
Have challenges in achieving adequate stable fly control and/or complying with national legal requirements on its control		□		

^a^www.basis-reg.co.uk

Table 6 lists success factors, challenges, and recommendations for other supply chain actors that were provided by these respondents. Several of the methods mentioned are in relation to pineapple foliage treatment alternatives. However, these methods are of wide relevance to other crops and paraquat uses. A summary of experiences from all supply chain respondents in terms of how they implemented paraquat phaseout is given in Supplementary Table S3.

**Table 6:** A summary of success factors, challenges, and recommendations on paraquat phaseout in supply chains that were described during consultations with supply chain stakeholders (sources: survey responses from 7 supply chain stakeholders, i.e., 4 retailers, 2 ISEAL members, one producer company, 6 were in relation to Costa Rican pineapple, and 1 in relation to coffee. Dec. 2021–Mar. 2022)

Success factors:
* • Farmer awareness raising*, *practical training*, *and learning from experiences of pioneering farmers are critical to successful phaseout*^a^ * • Changes in practice need investment, focused attention, and collaboration with others* * •* Raising the issue with suppliers and sharing of best practice at an early stage is key * •* A clear policy position on HHP use from the retailer is important so that new/existing suppliers are aware * •* Trusting supplier partnerships are important
Challenges:
* • Farmers’ access to knowledge*, *alternatives*, *and finance may be limited* * •* Some growers are resistant to changing their agronomic practices * •* Access to alternatives (e.g., decomposer microbes for Costa Rican pineapple) can be limited * •* Poor government policies which do not support HHP phaseout in practice * •* Agrochemical dealers influence farmer’s decision-making—for some farmers, these outlets are their only source of advice * •* Current supply chain data system does not collect detailed data on IPM strategies used to replace paraquat, so it is hard to identify successful practices or work on improvement * •* Retailer companies that demand progress on pesticide reduction but are not prepared to pay suppliers a little more for the effort and investment needed
Recommendations provided by respondents for other supply chain actors:
* • Growers tend to want to see how alternatives work in practice before adopting them.* Field visits and demonstration plots are key to convincing them and access to local positive experiences encourages them to implement these alternatives * •* It is important to include IWM in farmer training to reduce overall reliance on herbicides, rather than just replacing one herbicide with another * •* Supplier/grower working groups are an asset to discuss and review plans on a joint strategy to phase out paraquat * •* Sharing information on mitigation and phase out plans among the supply chain helps to build momentum * •* Growers need to be well informed of which biological alternatives can be used and how to apply them effectively * •* A company vision of zero pesticide residues and maximizing natural control methods motivates growers to buy into the concept of phasing out paraquat, and it can also help to access rewarding markets

^a^Factors in *italics* were reported by 3 or more respondents

### Lessons learnt from a regulator’s perspective

Seven current/former national pesticide regulators from countries that had banned paraquat in Europe, S. America, Asia, and the Pacific were consulted and given a pre-structured questionnaire that included questions on the implementation of national paraquat bans in their country along with three belief statements that were rated on a five-point Likert scale. The five response options were as follows: 1, strongly agree; 2, agree; 3, not sure; 4, disagree; and 5, strongly disagree. A summary of their responses is provided in the following section.

Reasons for national paraquat bans included both health and environmental harms, with paraquat related suicides, high toxicity, and lack of antidote frequently mentioned (Table 7). A combination of data sources were used to make an informed decision and justify the ban (or withdrawal). These mostly included national datasets along with workshops/consultations, but scientific data from international organizations and other countries were also used. Importantly, a wide range of stakeholders were commonly involved in the process to implement and then communicate the ban. In most cases, growers received support to transition away from paraquat via training and demonstrations of alternative methods by government agricultural extension services. In some cases, additional support was not needed due to pre-existing knowledge and availability of alternatives. Examples of successful processes and challenges in banning paraquat, along with recommendations for the future implementation of paraquat bans in other countries, are provided in Table 8.

**Table 7:** A summary of responses provided by seven current/former pesticide regulators on the reasons behind paraquat bans in their respective countries and the processes/stakeholders involved during the implementation of the ban

Reasons for ban	Data used	Justification	How was the ban and phase out communicated to growers?	How were growers supported to transition away from paraquat?	Stakeholders involved
-Paraquat-related suicides -Highly toxic -No antidote -Long-term health effects -Environmental effects -Poisoning of domestic pets	-Overall pesticide suicide data -Paraquat suicide data -Paraquat use -Hospital admission data -Scientific evidence (including those provided by international organizations and other countries)	-Following consultations and workshops, the conclusion was drawn that only a ban could be effective to avoid deaths from paraquat -The rate of death was significantly higher than the other pesticide formulations of WHO class II that were available -Information on the harmful effects of paraquat on people, animals, ecosystems, and the environment, including suicide cases	-Via agriculture ministries, government, extension services, and demonstration plots -Training agri-input suppliers -Public awareness raising/media campaigns -Posters/leaflets giving advice on the ban and on herbicide and non-herbicide alternatives (including posting in posters in agri-dealer shops) -Via pesticide industry networks -Via farmers’ organisations -Via website and letter to approval holders	-Training of agricultural extension officers and farmers in alternatives by Department of Agriculture -Field work, advising, and training farmers on how to manage weeds, select appropriate herbicides, get effective use, and monitoring for paraquat in shops -Strengthening extension services, recruiting more staff, and improving presence in rural areas -Farmers were informed of alternative pesticides to paraquat and demonstrations of the effectiveness of the recommended alternatives	-Ministry of Health -Ministry of Agriculture (both extension and research) -Palm oil/Cocoa/Pineapple Boards -National Poison Centre -Non-government organisations -Pesticide industry (manufacturers, registrants, sellers) -Farmers’ associations -Local community organizations

**Table 8:** Examples of successful processes and of challenges in banning paraquat described by seven current/former pesticide regulator respondents, along with their recommendations for the future implementation of paraquat bans in other countries

Successful processes:
• Training growers in the use of alternatives • Demonstrating the use of spray shields for taro as a method to protect vulnerable crops from alternative herbicides with systemic action • Enlisting border control and customs and setting heavy fines for illegal imports The creation of an “Alternative to Paraquat Committee: comprising relevant public and private sector members • An assertive awareness program was conducted in collaboration with various stakeholders to educate growers on how to use an integrated approach for the use of safer alternatives • Relevant stakeholders were engaged in developing national policy on paraquat. Successful policies were formulated by an independent advisory panel to the pesticide regulator, consisting of experts from health, agriculture, environment, customs, and occupational health established under the law governing pesticide control. The law provided necessary provisions for the control of import, distribution, marketing, sale and use of pesticides • Information about the government’s decision to remove paraquat, along with information on the toxicity of paraquat to human health, the reproductive system, and the environment was widely disseminated through various government channels, involving agricultural, health, and environmental departments and via mass media agencies • Farmers’ access to information was strengthened by an information system disseminated via village’s organizations such as village management board, Farmers’ Union, Women’s Union, Youth Union, and village health care services
Challenges:
• Management of illegal smuggling of paraquat from neighboring countries where paraquat is still legally sold or produced • Inadequate bans that allow registration of paraquat via alternative formulations • Limited stocks of alternative herbicides • Insufficient time to evaluate alternative methods before the ban was implemented due to lack of a transition period • Difficulty of convincing farmers because they do not view health and safety as a priority concern; they often appear more concerned with the cost and quick effectiveness of paraquat (a transition time allows for alternatives to be evaluated and demonstrated to farmers)
Recommendations made by regulators consulted:
• It is important to establish a credible and strong scientific evidence-based process in reviewing and decision-making • Throughout the banning process, pesticide regulators should work closely with the health sector, regularly review and make improvements to the decision-making process based on the feedback from the field, and coordinate closely with the pesticide industry • Regulators should consult with stakeholders before a ban, including, farmers, pesticide sellers, and pest management experts • A transition period is recommended before a full ban is implemented so as to give sufficient time to conduct research on alternatives and to train farmers • It is essential to convince farmers to use safer alternatives and encourage non-herbicide methods • Regulators have a responsibility to act to prevent suicides from paraquat. The most effective action is to ban paraquat

Of the seven current/former regulators who addressed the following three belief statements, all agreed that, to their knowledge, “*banning paraquat had no adverse effect on crop yields*” (Supplementary Table S4). Four out of seven respondents considered that “*banning paraquat has had no adverse effect on farmer incomes*” while the remaining three respondents were unsure due to a lack of knowledge. Five of seven agreed that “*banning paraquat has greatly reduced incidences of human pesticide poisonings*.” The remaining two respondents were “not sure” due to the short interval since the ban and therefore lack of data.

## Discussion

We highlight in this review that paraquat poses a severe health hazard to humans and the environment, over 67 countries have banned paraquat, at least three well-known PVS active in LMICs have prohibited use of paraquat by their produce suppliers, and several European retailers have achieved, or are working towards, zero use across their supply chains, without major reductions in agricultural yield or increases in costs. Peer-reviewed literature is lacking on practical and economic studies of alternatives for paraquat. However, the reality that paraquat has been successfully banned across so many countries and supply chains demonstrates that alternatives to paraquat do exist across a number of crops and environments. This is supported by our synopsis of less hazardous and more sustainable alternatives to paraquat, along with crop-specific case studies and lessons learnt from phasing out this HHP herbicide.

There are understandable concerns among national pesticide regulators, farmers, and the agricultural and trade sectors about possible negative economic and food production consequences from banning paraquat. However, the experiences shared by regulators and supply chains consulted in this study failed to find evidence of such consequences. Following a review of the literature and an analysis of available data from FAOSTAT, we found no evidence across any of the countries assessed to suggest that a ban on paraquat had negative effects on crop yields at a national or state level. This finding was supported by regulators from countries that had banned paraquat who were consulted for this paper. More than half of those consulted agreed that banning paraquat had no adverse effect on farmer incomes. The remaining 43% were not sure due to lack of knowledge. In addition, our findings highlight that major uses of paraquat extend beyond weed management and minimizing yield loss to tasks such as improving the efficiency of the harvest process and post-harvest foliage waste removal. Not only do these uses have little to do with crop productivity but some introduce an added food safety concern for consumers due to paraquat application in food crops close to harvest.

Our review of the literature along with the documented experiences shared by regulators and supply chains show that there are numerous alternative methods for managing weeds or removing crop foliage that can successfully replace paraquat. These include non-herbicide and herbicide methods. However, caution should be applied when selecting substitute herbicides to replace paraquat for a number of reasons: (i) substituting paraquat with other widely available herbicides, e.g., diquat, glyphosate, or glufosinate-ammonium, may replace one set of hazards with different ones. Glyphosate, glufosinate-ammonium, and other popular herbicides qualify as HHPs for chronic human health hazards, dietary risk to consumers, environmental toxicity, and/or environmental persistence. Diquat, a close relative of paraquat, is of particular concern as a replacement because it is highly toxic to humans and poses higher risk than paraquat to natural enemies of pests (Table 2); (ii) some herbicides may not be suitable replacements due to their systemic action that can destroy the crop or cause sub-lethal phytotoxicity and (iii) the risk of developing weed resistance to the substitute herbicide (glyphosate in particular). Because of such concerns, along with a greater awareness of the harms caused by herbicides and the benefits of weeds, there is consensus building among weed scientists that we need to move away from current levels of herbicide reliance and rethink approaches to managing/living with a level of weeds and better understand their benefits (Merfield 2019).

### Lessons learnt to support regulatory action

The responses provided by regulators consulted in this study highlighted that reasons for national paraquat bans included both health and environmental harms, with paraquat related suicides, high toxicity, and lack of an antidote mentioned by several respondents. These reasons are in line with official notifications to the Rotterdam Convention, which identified widespread incidents of acute health effects among spray operators using paraquat (CRC 2019). For example, in their official notification, CILSS countries identified known hazards and risks of paraquat to be “high acute toxicity,” “unacceptable risk of intoxication for users in the Sahelian conditions of use,” and “high toxicity to birds.” Malaysia identified “high toxicity,” “lack of antidote,” and “long-term and delayed health effects” as known hazards and risks; it also reported that “paraquat is highly used for suicidal purposes.” In Mozambique’s notification to the Convention’s Chemical Review Committee (CRC), an assessment was provided based on a thorough review of paraquat use conditions that concluded that it is unlikely that locally feasible mitigation measures would reduce the risk of paraquat to acceptable levels. An occupational risk assessment of six HHPs, including paraquat, at registered dose rates, both with and without PPE, revealed that acceptable operator exposure levels (AOEL) would be exceeded in all cropping systems assessed.

The pesticide regulators consulted for this paper shared several common success factors for banning paraquat, including (i) the establishment of an independent and multi-stakeholder advisory panel or committee early in the process, including agriculture, health and environment interests, (ii) engaging with different public and private sector organisations, including customs departments to plan and coordinate actions, and (iii) awareness programs and information sharing, in collaboration with various stakeholders, about paraquat hazards, planned bans, restriction and transition periods, and to promote alternative methods to producers, especially on how to use an IWM approach. Sharing science-based evidence on paraquat’s risks to human health and the environment, as well as the alternatives available, is important to increase public support and to help farmers to understand the health protection rationale for bans (WHO 2021b).

A number of challenges were also mentioned. One of these was that in spite of restrictions in place, illegal trade across borders has allowed the continued use of paraquat. This has previously been reported in several countries following paraquat bans, such as Laos and French Guiana, leading to a continuation of paraquat poisoning incidents occurring (Flechel et al. 2018, Rassapong et al. 2018, Siodmak 2016). Laos government officials previously considered it a challenge to implement pesticide regulations because of the country’s long, porous borders with pesticide manufacturing countries such as China and Vietnam (Vazquez 2013). China and Vietnam’s recent decisions to ban paraquat may thus help reduce the availability of paraquat in Laos. However, in China, paraquat production for export is still permitted. The difficulty of controlling transboundary movement of banned pesticides provides a strong argument for the introduction of regional bans on paraquat, as has been implemented by CILSS and EU countries, or even a global ban. A regional or global ban could also help reduce address the fear of imbalances in international agricultural competitiveness following a national ban. For example, an economic modelling study by Walsh and Kingwell (2021) concluded that if both paraquat and glyphosate were to be banned in Australia, Australian grain farming with glyphosate-tolerant crops would be at a competitive disadvantage in a global market, but a wider or global ban of these HHPs would allow Australian grain farmers to remain economically competitive. However, it should be noted that this study was limited in the scope of alternative weed management approaches assessed, and the authors rightly acknowledged that a decline in profit is a common result of changing farm practices but can actually stimulate innovation in weed management at both the farm and industry level so as to reduce costs. We should also emphasize that to avoid further delay in saving lives, governments should not wait for global action.

Another challenge lies in convincing farmers of the health risks and need to consider alternative approaches to paraquat use. This re-emphasizes the need to liaise closely with the agricultural sector and farmers’ organisations to help farmers and extension agents fully understand the health protection rationale for bans and build their knowledge and confidence in using IWM and alternative methods. Farmer awareness raising, practical training, and learning from experiences are important for successful phaseout of HHPs such as paraquat.

Two review studies by Moss (2019) and Tataridas et al. (2022) provide further insights into why farmers are reluctant to use non-herbicide alternatives and IWM along with suggestions for approaches that could lead to greater uptake. Examples include the need to include participation of farmers in the decision-making process, paying more consideration to the farmer’s perspective and a better understanding of the factors influencing farmer behavior. We advocate presenting advice in a manner that coincides with the farmer’s experiences and attitudes and changing farmers’ weed control “mindset” from one based primarily on short-term herbicide solutions to one based on longer term, more diverse vegetation management strategies.

Two further challenges identified by regulators in this study included the limited availability of stocks of alternative herbicides following a ban and insufficient time to evaluate alternative methods before the ban was implemented due to lack of a transition period. Both of these point to the benefits of a limited transition period following the announcement of the ban as was recommended by both regulators and PVS respondents in this study. This is supported by the well-documented success story of the paraquat ban in Sri Lanka, during which a three-year phase-out program was conducted (Gunnell et al. 2017; Marambe and Herath 2019). This began in 2008 with the introduction of a 6.5-g L^–1^ paraquat ion formulation to replace the 200-g L^–1^ formulation, along with phased import bans until the complete ban was imposed in 2011. However, the benefits of a transition period must be weighed against serious ethical concerns regarding continued use of a herbicide that is linked to significant harm to human health.

### Lessons learnt to support the supply chain sector

Following the consultation with supply chain actors, the key points that emerged were that phasing out paraquat requires (i) time, (ii) investment, especially in farmer awareness raising of the rationale for phaseout, and (iii) practical training on alternatives. Testing alternatives and refining how different IWM methods can be combined for farmers in different agronomic contexts and areas is important as there are unlikely to be “one size fits all” solutions within one country, even in the same cropping system. The process also benefits hugely from problem- and solution-focused grower group discussions and learning forums with agronomists. Access to local positive experiences and pioneer farmer knowledge provides inspiration and encourages other farmers to implement these changes. In terms of time scale, some relatively quick phaseouts have been achieved—two Costa Rican respondents reported that alternatives for pineapple foliage destruction have taken about three years to put into place and refine, while in Vietnam, national phaseout of paraquat in coffee was achieved within a two-year transition period.

As with regulators, PVS suppliers consulted emphasized that close and constructive collaboration between relevant stakeholders is another essential factor for success. Such collaboration can lead to important co-benefits or added value, e.g., in Vietnam, experiences developed in phasing out paraquat with different weed management alternatives put the coffee sector in a good position to then work on glyphosate reduction. Policy advocacy by the national chapter of the Global Coffee Platform convinced the regulators to take the decision to withdraw approval of glyphosate in coffee, within just 18 months.

## Conclusions

We conclude that eliminating paraquat will save lives without reducing agricultural production. Less hazardous and more sustainable alternatives exist. Successful adoption and uptake of these methods at scale involves farmer training and support, along with an enabling policy environment. Government policies in general are needed to support the aims of reducing harms from herbicides by reducing reliance on them, with concerted actions on phasing out paraquat as a priority.

Unfortunately, although paraquat has been banned in over 67 countries, it is still widely used in many countries. There is a paucity of published data on practical and economic assessments of alternatives to paraquat. We hope that this analysis goes some way to filling that gap. Our review shows that a wide range of alternative approaches to weed management and foliage removal are available, many of which do not rely on chemical herbicides. Our findings provide evidence that paraquat phaseout and replacement is both technically and economically feasible. For long-term sustainability of the agro-ecosystem, it is vital that IWM methods are adopted for weed management, with a focus on non-herbicide methods. Their effective implementation requires collaborative action by government agencies, producer groups, weed scientists and agronomists and food and fiber supply chains and retailers.

## Supplementary Information

Below is the link to the electronic supplementary material.

Supplementary file1 (DOCX 51 KB)

## Data Availability

Data and materials for this study are available and will be provided to the journal upon request.
