# Supplementary material for: Agriculture without paraquat is feasible without loss of productivity—lessons learned from phasing out a highly hazardous herbicide
Source: Environ Sci Pollut Res Int. 2023 Jan 9;30(7):16984–7008. doi: 10.1007/s11356-022-24951-0 (PMC9928820; doi:10.1007/s11356-022-24951-0)
Supplement: Supplementary file 1 — Supplementary file1 (DOCX 51 KB) [file 11356_2022_24951_MOESM1_ESM.docx]

**Supplementary files**

**Title:** Agriculture without paraquat is feasible without loss of productivity. Lessons learned from phasing out a highly hazardous herbicide

**Journal:** Environmental Science and Pollution Research

**Authors:** Alexander M. Stuart^a^, Charles N. Merfield^b^, Finbarr G. Horgan^cde^, Sheila Willis^a^, Meriel A. Watts^f^, Fernando Ramírez-Muñoz^g^, Jorge Sánchez U^h^, Leah Utyasheva^c^, Michael Eddleston^c^, Mark L. Davis^c^, Lars Neumeister^i^, Manoé R. Sanou^j^, Stephanie Williamson^a^

^a^Pesticide Action Network UK, Brighthelm Centre, Brighton, UK

^b^Merfield Agronomy Ltd., Lincoln, New Zealand

^c^Centre for Pesticide Suicide Prevention, University of Edinburgh Edinburgh, UK

^d^Escuela de Agronomía, Facultat de Ciencias Agrarias y Forestales, Universidad Católica del Maule, Casilla 7-D, Curicó 3349001, Chile

^e^EcoLaVerna Integral Restoration Ecology, Bridestown, Kildinan, T56 P 499, Cork, Ireland

^f^PAN Asia Pacific, Penang, Malaysia

^g^Central American Institute for Studies on Toxic Substances (IRET), Universidad Nacional, Costa Rica

^h^Nicoverde S.A., Costa Rica

^i^Pesticide Expert, Germany

^j^Department of Plant Protection and Packaging, Ministry of Agriculture, Burkina Faso

**Corresponding author:** Alexander M. Stuart, [alex@pan-uk.org](mailto:alex@pan-uk.org)

**Supplementary information sources for Table 3**

**BCI Principles and Criteria**. version 2.1 | 1 March 2018. Via: <https://bettercotton.org/what-we-do/defining-better-our-standard/> *Accessed 17/01/22*

**Bonsucro Production Standard** version 4.2 Dec. 2016. Via: <https://bonsucro.com/production-standard/> *Accessed 17/01/22*

**Fairtrade Standard for Small-scale Producer Organisations**, version 03.04.2019_v2.5, 2019. Via: <https://www.fairtrade.net/standard> *Accessed 05/05/22*

**Fairtrade International Hazard Materials List**, version 1.12.2016 v 1.4. Via: <https://www.fairtrade.net/standard/fairtrade-standards#hml> *Accessed 17/01/22*

**FSC International Standard. FSC Principles and Criteria for Forestry Stewardship.** Version FSC-STD-01-001 V5-2 EN, 2015. Via: https://fsc.org/en/document-centre/documents/resource/392 *Accessed 05/05/22*

**FSC List of highly hazardous pesticides**, FSC-POL-30-001a EN version 2019. Via: <https://fsc.org/en/document-centre/documents/resource/315>. *Accessed 17/01/22*

**Global Coffee Platform Coffee Sustainability Reference Code** version 3.0 2021. Via: <https://www.globalcoffeeplatform.org/our-work/coffee-sustainability-reference-code/> *Accessed 05/05/22*

**Global Coffee Platform Coffee Sustainability Reference Code, Annex Pesticides List**. Via: <https://www.globalcoffeeplatform.org/wp-content/uploads/2021/10/CSRC_PesticidesLists_OCT21.pdf> *Accessed 05/05/22*

**IPM Coalition: Paraquat dichloride.** Via: <https://ipm-coalition.org/term/10729/paraquat-dichloride>. *Accessed 17/01/22*

**Rainforest Alliance Sustainable Agriculture Standard, Farm Requirements**, version SA-S-SD-1-V1.2, 2022 Via: <https://www.rainforest-alliance.org/for-business/2020-certification-program/#standard> *Accessed 05/05/22*

**Rainforest Alliance Annex S7. Pesticides Management**, version 1.2, 2021. *Via:* [*https://www.rainforest-alliance.org/wp-content/uploads/2020/06/Annex-7-Pesticides-Management.pdf*](https://www.rainforest-alliance.org/wp-content/uploads/2020/06/Annex-7-Pesticides-Management.pdf) *Accessed 05/05/22*

**RSPO Principles and Criteria for the Production of Sustainable Palm Oil.** Version 2018* with 2020 update. Via: <https://rspo.org/resources/archive/1440> *Accessed 17/01/22*

**RTRS Standard for Responsible Soy Production** Version 4.0. Via: <https://responsiblesoy.org/wp-content/uploads/2021/12/RTRS-Standard-for-Responsible-Soy-Production-V4.0.pdf>. *Accessed 17/01/22*

**Supplementary Table S1.** List of countries that have banned or withdrawn the registration of paraquat and the years in which this was implemented, along with known phase out (transition) periods

|  | **Countries with paraquat bans** | **Year full ban implemented** | **Year phase out started** | **Source** |
| --- | --- | --- | --- | --- |
| 1 | Kuwait | 1985 | - | Watts M 2011: Paraquat, Pesticide Action Network Asia and the Pacific (PANAP); Cooperation Council for the Arab States of the Gulf Secretariat (2015) General Pesticides Law (System) and its Implementing Regulation in the Gulf Cooperation. |
| 2 | Norway | 1995 | - | Norway database on registered plant protection products (2022). Available at: https://www.mattilsynet.no/plantevernmidler/godk.asp?sortering=preparat&preparat=Alle&sprak=In+English (Accessed 23/07/22) |
| 3 | Switzerland | 2002 | - | PAN (2022) Consolidated List of Banned Pesticides. Pesticide Action Network (PAN) International. Available at: https://pan-international.org/pan-international-consolidated-list-of-banned-pesticides/ (Accessed 23/07/22) |
| 4 | Cambodia | 2003 | - | PAN (2022) Consolidated List of Banned Pesticides. Pesticide Action Network (PAN) International. Available at: https://pan-international.org/pan-international-consolidated-list-of-banned-pesticides/ (Accessed 23/07/22) |
| 5 | Côte d'Ivoire | 2004 | - | Watts M 2011: Paraquat, Pesticide Action Network Asia and the Pacific (PANAP); CILSS (2011): Paraquat ban [in French]. Decision No.125/COOR/2011. . Permanent Inter-State Committee for Drought Control in the Sahel (CILSS) |
| 6 | Malaysia | 2005-2006, 2020 | 2003-2005 | PAN (2022) Consolidated List of Banned Pesticides. Pesticide Action Network (PAN) International. Available at: https://pan-international.org/pan-international-consolidated-list-of-banned-pesticides/ (Accessed 23/07/22) |
| 33 | EU (27 countries) | 2007-2008 | 2007 | PAN (2022) Consolidated List of Banned Pesticides. Pesticide Action Network (PAN) International. Available at: https://pan-international.org/pan-international-consolidated-list-of-banned-pesticides/ (Accessed 23/07/22) |
| 34 | UK | 2008 | 2007 | PAN (2022) Consolidated List of Banned Pesticides. Pesticide Action Network (PAN) International. Available at: https://pan-international.org/pan-international-consolidated-list-of-banned-pesticides/ (Accessed 23/07/22) |
| 35 | Serbia | 2009 | - | Official Gazette of the Republic of Serbia (2009). 41/09 |
| 36 | South Korea | 2011 | - | PAN (2022) Consolidated List of Banned Pesticides. Pesticide Action Network (PAN) International. Available at: https://pan-international.org/pan-international-consolidated-list-of-banned-pesticides/ (Accessed 23/07/22) |
| 37 | Lao PDR | 2011 | - | PAN (2022) Consolidated List of Banned Pesticides. Pesticide Action Network (PAN) International. Available at: https://pan-international.org/pan-international-consolidated-list-of-banned-pesticides/ (Accessed 23/07/22) |
| 38 | Burkina Faso | 2011 | - | CILSS (2011): Paraquat ban [in French]. Decision No.125/COOR/2011. . Permanent Inter-State Committee for Drought Control in the Sahel (CILSS) |
| 39 | Cabo Verde | 2011 | - | CILSS (2011): Paraquat ban [in French]. Decision No.125/COOR/2011. . Permanent Inter-State Committee for Drought Control in the Sahel (CILSS) |
| 40 | Chad | 2011 | - | CILSS (2011): Paraquat ban [in French]. Decision No.125/COOR/2011. . Permanent Inter-State Committee for Drought Control in the Sahel (CILSS) |
| 41 | Gambia | 2011 | - | CILSS (2011): Paraquat ban [in French]. Decision No.125/COOR/2011. . Permanent Inter-State Committee for Drought Control in the Sahel (CILSS) |
| 42 | Guinea-bissau | 2011 | - | CILSS (2011): Paraquat ban [in French]. Decision No.125/COOR/2011. . Permanent Inter-State Committee for Drought Control in the Sahel (CILSS) |
| 43 | Mali | 2011 | - | CILSS (2011): Paraquat ban [in French]. Decision No.125/COOR/2011. . Permanent Inter-State Committee for Drought Control in the Sahel (CILSS) |
| 44 | Mauritania | 2011 | - | CILSS (2011): Paraquat ban [in French]. Decision No.125/COOR/2011. . Permanent Inter-State Committee for Drought Control in the Sahel (CILSS) |
| 45 | Niger | 2011 | - | CILSS (2011): Paraquat ban [in French]. Decision No.125/COOR/2011. . Permanent Inter-State Committee for Drought Control in the Sahel (CILSS) |
| 46 | Palestine, State of | 2011 | - | PAN (2022) Consolidated List of Banned Pesticides. Pesticide Action Network (PAN) International. Available at: https://pan-international.org/pan-international-consolidated-list-of-banned-pesticides/ (Accessed 23/07/22) |
| 47 | Senegal | 2011 | - | CILSS (2011): Paraquat ban [in French]. Decision No.125/COOR/2011. . Permanent Inter-State Committee for Drought Control in the Sahel (CILSS) |
| 48 | Sri Lanka | 2012 | 2009 | PAN (2022) Consolidated List of Banned Pesticides. Pesticide Action Network (PAN) International. Available at: https://pan-international.org/pan-international-consolidated-list-of-banned-pesticides/ (Accessed 23/07/22) |
| 49 | Mozambique | 2014 | - | PAN (2022) Consolidated List of Banned Pesticides. Pesticide Action Network (PAN) International. Available at: https://pan-international.org/pan-international-consolidated-list-of-banned-pesticides/ (Accessed 23/07/22) |
| 50 | Oman | 2014 | - | Cooperation Council for the Arab States of the Gulf Secretariat (2015) General Pesticides Law (System) and its Implementing Regulation in the Gulf Cooperation. |
| 51 | Turkey | 2014 | 2013 | PAN (2022) Consolidated List of Banned Pesticides. Pesticide Action Network (PAN) International. Available at: https://pan-international.org/pan-international-consolidated-list-of-banned-pesticides/ (Accessed 23/07/22) |
| 52 | Bahrain | 2015 | - | Cooperation Council for the Arab States of the Gulf Secretariat (2015) General Pesticides Law (System) and its Implementing Regulation in the Gulf Cooperation. |
| 53 | Saudi Arabia | 2015 | - | Cooperation Council for the Arab States of the Gulf Secretariat (2015) General Pesticides Law (System) and its Implementing Regulation in the Gulf Cooperation. |
| 54 | Togo | 2015 | - | PAN (2022) Consolidated List of Banned Pesticides. Pesticide Action Network (PAN) International. Available at: https://pan-international.org/pan-international-consolidated-list-of-banned-pesticides/ (Accessed 23/07/22) |
| 55 | United Arab Emirates | 2015 | - | Cooperation Council for the Arab States of the Gulf Secretariat (2015) General Pesticides Law (System) and its Implementing Regulation in the Gulf Cooperation. |
| 56 | Qatar | 2015 | - | Cooperation Council for the Arab States of the Gulf Secretariat (2015) General Pesticides Law (System) and its Implementing Regulation in the Gulf Cooperation. |
| 57 | Taiwan | 2018 | - | PAN (2022) Consolidated List of Banned Pesticides. Pesticide Action Network (PAN) International. Available at: https://pan-international.org/pan-international-consolidated-list-of-banned-pesticides/ (Accessed 23/07/22) |
| 58 | Malawi | 2019 | - | Pesticide Act (2019) The Malawi Government Gazette. 56 (11). |
| 59 | Vietnam | 2019 | 2017 | PAN (2022) Consolidated List of Banned Pesticides. Pesticide Action Network (PAN) International. Available at: https://pan-international.org/pan-international-consolidated-list-of-banned-pesticides/ (Accessed 23/07/22) |
| 60 | Brazil | 2020 | 2017 | PAN (2022) Consolidated List of Banned Pesticides. Pesticide Action Network (PAN) International. Available at: https://pan-international.org/pan-international-consolidated-list-of-banned-pesticides/ (Accessed 23/07/22) |
| 61 | China | 2020 | - | PAN (2022) Consolidated List of Banned Pesticides. Pesticide Action Network (PAN) International. Available at: https://pan-international.org/pan-international-consolidated-list-of-banned-pesticides/ (Accessed 23/07/22) |
| 62 | Fiji | 2020 | - | PAN (2022) Consolidated List of Banned Pesticides. Pesticide Action Network (PAN) International. Available at: https://pan-international.org/pan-international-consolidated-list-of-banned-pesticides/ (Accessed 23/07/22) |
| 63 | Thailand | 2020 | - | Government of Thailand (2020) Ministry of Industry 2020. Notification B.E. 2563 (2020) re: List of Hazardous Substances (Issue No. 6) [in Thai]. The Royal Gazette 137(117). |
| 64 | Morocco | 2021 | - | PAN (2022) Consolidated List of Banned Pesticides. Pesticide Action Network (PAN) International. Available at: https://pan-international.org/pan-international-consolidated-list-of-banned-pesticides/ (Accessed 23/07/22) |
| 65 | Maldives | 2022 | - | Rotterdam Convention Notifications of Final Regulatory Action - Non-Annex III Chemicals. Available at: http://archive.pic.int/viewB_FRAchems.php?player=198 (Accessed 02/07/22) |
| 66 | Peru | 2022 | - | PAN (2022) Consolidated List of Banned Pesticides. Pesticide Action Network (PAN) International. Available at: https://pan-international.org/pan-international-consolidated-list-of-banned-pesticides/ (Accessed 23/07/22) |
| 67 | Benin | - | - | CILSS (2011): Paraquat ban [in French]. Decision No.125/COOR/2011. . Permanent Inter-State Committee for Drought Control in the Sahel (CILSS) |
| 68 | Guinea | - | - | CILSS (2011): Paraquat ban [in French]. Decision No.125/COOR/2011. . Permanent Inter-State Committee for Drought Control in the Sahel (CILSS) |

**Supplementary Table S2.** Summary of paraquat-resistant weed records from the International Herbicide Resistant Weeds database by geographical region (Source: www.weedscience.org)

| **Region** | **Countries and no. individual records** | **No, resistance records per region** | **No. of weed species recorded resistant per region** | **Crop-specific resistance records for two problematic weeds**  **Goosegrass (GG) and Fleabanes (FB)** |
| --- | --- | --- | --- | --- |
| Africa | Kenya  Egypt  South Africa x 3 | **5** | **3** | FB *C. bonariensis:* grapes; orchards |
| Asia-Pacific | China x 5  Fiji  Indonesia x 2  Japan x 7  Malaysia x 9  Sri Lanka  Taiwan | **25** | **17** | GG: vegetables (Malaysia); oil palm (Indonesia); maize (Indonesia); unspecified crops (China)  FB *C. canadensis:* almonds; roadsides (Japan)  FB *C. sumatrensis:* orchards; tea; vegetables; roadsides; unspecified crops |
| Australasia | Australia x14  New Zealand x 2 | **16** | **11** | GG: sugarcane (Australia)  FB *C. sumatrensis*: wheat; fallows (Australia)  FB *C. bonariensis:* grapes ((Australia) |
| Europe | Belgium x 3  UK x 2 | **5** | **3** | FB *C. canadensis*; nurseries |
| Middle East | Jordan | **1** | **1** |  |
| North America | Canada x 3  USA x 11 | **14** | **8** | GG: tomato (US)  FB *C. canadensis*: almonds; peaches; soybean  FB *C. bonariensis:* orchards; roadsides (California) |
| South & Central America | Brazil x 3  Colombia  Paraguay | **5** | **2** | GG: maize (Colombia)  FB *C. sumatrensis*: soybean |

|  | **Supplementary Table S3.** Synopsis of supply chain activities to implement paraquat phase out as reported by UK retailer and PVS respondents.   \| **Respondent** \| **How were growers supported to transition away from paraquat?** \| **Stakeholders involved** \| **Progress to date** \| \| --- \| --- \| --- \| --- \| \| UK retailer A \| Facilitate pineapple grower discussions on alternatives via supply chain specific action group, helping to support best practice and peer-to-peer learning. \| Supply chain staff, qualified agronomists, growers \| On-going work to reduce and phase out use. Growers must justify clearly why they still find it necessary to use and detail any use of relevant IPM methods. Requests for paraquat use have declined since work to explore non-chemical alternatives. Alternative methods based on mechanical destruction and incorporation seem to be the most widely adopted. Growers currently looking at ways to improve and enhance performance of alternatives. \| \| UK retailer B \| On-going work to complete phase out, by derogation only, with numerous restrictions. \| No info \| Massive reduction in last few years, usage is only in rare and particular cases. \| \| UK retailer C \| -Set up a suppliers’ crop protection group in 2021 to begin implementing company policy on HHP phase out and IPM replacement.  -Posting info on alternative practices on supply chain wide platform for pineapple growers.  -Work with crop-specific suppliers to identify where HHPs are used, then jointly trial alternatives. \| Company, suppliers’ working group representatives, leading growers/exporters \| Use discontinued by one major supplier in 2019, by shredding, decomposer microbes and re-incorporation of rotted material. This experience provides best practice example for others to follow. \| \| UK retailer D \| No info \| No info \| Paraquat application for pineapple foliage desiccation discontinued by growers some years ago \| \| PVS 1 \| Dedicated training for pineapple growers on alternatives from 2018, as part of usual training to comply with the requirements of this supply chain. \| Supply chain staff and trainers, pioneer farmers using alternatives \| None of the pineapple growers are using paraquat. Most are now using decomposer microbes, although effectiveness varies. \| \| PVS 2 (Coffee) \| Collective action initiatives set up by PVS in collaboration with grower groups and Ministry of Agriculture. Close liaison with pesticide regulators on phase out dates and transition periods for bans on paraquat, glyphosate. \| Growers, commodity board, supply chain standard, Ministry of Agriculture \| Paraquat no longer used in coffee, replaced by hand or mechanical weeding and use of other herbicides. Some use of organic/biological herbicides. \|   **Supplementary Table S4.** The extent of agreement or disagreement for each of the statements below that were provided by regulator respondents. 1-strongly agree, 2-agree, 3-not sure, 4-disagree, or 5-strongly disagree | | | | | | | | |
| --- | --- | --- | --- | --- | --- | --- | --- | --- | --- | --- | --- | --- | --- | --- | --- | --- | --- | --- | --- | --- | --- | --- | --- | --- | --- | --- | --- | --- | --- | --- | --- | --- | --- | --- | --- | --- | --- |
| **To my knowledge,** | | **Country A** | **Country B** | **Country C** | **Country D** | **Country E** | **Country F** | **Country G** | **% agree/ strongly agree** |
| banning paraquat has had no adverse effect on crop yields | | 2 | 1 | 2 | 1 | 1 | 2 | 2 | 100.0% |
| banning paraquat has had no adverse effect on farmer incomes | | 3 | 1 | 3 | 3 | 1 | 2 | 1 | 57.1% |
| banning paraquat has greatly reduced incidences of human pesticide poisonings | | 2 | 1 | 2 | 1 | 1 | 3 | 3 | 71.4% |

**Supplementary Figure S1.** Mean annual yield for previously paraquat dependent crops for three countries 6-7 years before and after paraquat was banned. Vertical line indicates year of ban. Source: FAOSTAT data accessed on 3^rd^ February 2022.
